# Exposure to ethanol leads to midfacial hypoplasia in a zebrafish model of FASD via indirect interactions with the Shh pathway

**DOI:** 10.1186/s12915-021-01062-9

**Published:** 2021-07-01

**Authors:** Alfire Sidik, Groves Dixon, Desire M. Buckley, Hannah G. Kirby, Shuge Sun, Johann K. Eberhart

**Affiliations:** 1 Department of Molecular Biosciences, Institute for Cellular and Molecular Biology, Institute for Neuroscience, Waggoner Center for Alcohol and Addiction Research, University of Texas at Austin, Austin, Texas 78712 USA; 2 Department of Integrative Biology, Institute for Cellular and Molecular Biology, University of Texas at Austin, Austin, Texas 78712 USA

**Keywords:** Wnt/planar cell polarity (PCP) pathway, Convergent extension, Vangl2, Sonic Hedgehog (Shh), Fetal alcohol spectrum disorders (FASD), Ethanol, Cyclopia, Polarity, Gastrulation

## Abstract

**Background:**

Gene-environment interactions are likely to underlie most human birth defects. The most common known environmental contributor to birth defects is prenatal alcohol exposure. Fetal alcohol spectrum disorders (FASD) describe the full range of defects that result from prenatal alcohol exposure. Gene-ethanol interactions underlie susceptibility to FASD, but we lack a mechanistic understanding of these interactions. Here, we leverage the genetic tractability of zebrafish to address this problem.

**Results:**

We first show that *vangl2*, a member of the Wnt/planar cell polarity (Wnt/PCP) pathway that mediates convergent extension movements, strongly interacts with ethanol during late blastula and early gastrula stages. Embryos mutant or heterozygous for *vangl2* are sensitized to ethanol-induced midfacial hypoplasia. We performed single-embryo RNA-seq during early embryonic stages to assess individual variation in the transcriptional response to ethanol and determine the mechanism of the *vangl2*-ethanol interaction. To identify the pathway(s) that are disrupted by ethanol, we used these global changes in gene expression to identify small molecules that mimic the effects of ethanol via the Library of Integrated Network-based Cellular Signatures (LINCS L1000) dataset. Surprisingly, this dataset predicted that the Sonic Hedgehog (Shh) pathway inhibitor, cyclopamine, would mimic the effects of ethanol, despite ethanol not altering the expression levels of direct targets of Shh signaling. Indeed, we found that ethanol and cyclopamine strongly, but indirectly, interact to disrupt midfacial development. Ethanol also interacts with another Wnt/PCP pathway member, *gpc4*, and a chemical inhibitor of the Wnt/PCP pathway, blebbistatin, phenocopies the effect of ethanol. By characterizing membrane protrusions, we demonstrate that ethanol synergistically interacts with the loss of *vangl2* to disrupt cell polarity required for convergent extension movements.

**Conclusions:**

Our results show that the midfacial defects in ethanol-exposed *vangl2* mutants are likely due to an indirect interaction between ethanol and the Shh pathway. Vangl2 functions as part of a signaling pathway that regulates coordinated cell movements during midfacial development. Ethanol exposure alters the position of a critical source of Shh signaling that separates the developing eye field into bilateral eyes, allowing the expansion of the midface. Collectively, our results shed light on the mechanism by which the most common teratogen can disrupt development.

**Supplementary Information:**

The online version contains supplementary material available at 10.1186/s12915-021-01062-9.

## Background

Birth defects manifest as structural or functional malformations at birth and are the leading cause of infant mortality in the USA [1]. Although the multifactorial etiologies of birth defects are not well understood, many are thought to derive from a complex interplay between genetic and environmental factors. The adverse effects of teratogens, or environmental agents that cause irreversible developmental defects, were first recognized in the 1950s and 1960s [2]. It was then that investigators began to observe developmental anomalies in infants exposed to methylmercury and thalidomide in utero [3, 4]. Since then, other environmental teratogens have come to light, including but not limited to, pollutants, pharmaceuticals, and chemicals.

Prenatal alcohol exposure (PAE) is the most common preventable cause of birth defects, yet the prevalence of fetal alcohol spectrum disorders (FASD) in the USA is as high as 2–5% [5]. Fetal alcohol syndrome (FAS) is the most severe outcome following PAE and is characterized by midfacial hypoplasia, as well as growth and neural deficits [6]. While PAE is required for the development of FASD, the teratogenic effects of ethanol are modulated by genetics [7, 8]. For instance, monozygotic twins were 100% concordant for FAS, whereas dizygotic twins were only 63% concordant [8, 9]. Furthermore, animal models of FAS show strain-specific differences after controlling for environmental variables such as dose and timing [10, 11]. Despite this, the genetic factors that protect or predispose an individual to FASD are poorly understood. Moreover, we still lack a basic understanding of the mechanism of ethanol teratogenesis. Zebrafish are well suited to address this problem due to their genetic tractability and ease of embryological manipulation [12].

In a screen to identify genetic modifiers of ethanol teratogenicity, *vang-like 2* (*vangl2*), a member of the non-canonical Wnt/planar cell polarity (Wnt/PCP) pathway, emerged as an ethanol sensitive locus [13]. In zebrafish, mutations in the *vangl2* locus disrupt convergent extension movements during gastrulation, resulting in a shortened, broadened body axis [14]. These mutants can present with cyclopia, a fusion of the bilateral eyes, but the phenotypic expressivity of this trait varies with factors such as temperature and genetic background [15]. In the screen, all untreated *vangl2* mutants displayed proper separation of the eyes and craniofacial skeletal elements were intact [13]. Upon exposure to 1% ethanol, a dose that does not normally cause facial defects, *vangl2* mutants were fully penetrant for cyclopia and displayed profound defects to the midfacial skeleton [13]. Ethanol-treated *vangl2* heterozygotes were largely indistinguishable from their wild-type siblings, with the exception of a single synophthalmic ethanol-treated heterozygote, providing evidence for latent haploinsufficiency. Together, these data suggest a synergistic interaction between a mutation in *vangl2* and ethanol.

We know *vangl2* plays a critical role in mediating convergent extension movements as evidenced by their body axis defect; however, because the early effects of ethanol exposure remain poorly defined, the precise mechanism of the *vangl2*-ethanol interaction remain elusive. To better understand how ethanol interacts with loss of *vangl2* to alter phenotypic outcomes, we took an unbiased approach to assess the transcriptional response to ethanol. We performed single embryo RNA sequencing (RNA-seq) on control (untreated) and ethanol-treated wild-type embryos in a time-course spanning gastrulation and early segmentation stages in zebrafish.

Bioinformatic and functional analyses indicated that midfacial defects in ethanol-exposed *vangl2* mutants were likely due to an indirect interaction between ethanol and the Shh pathway. While there was no alteration in the level of expression of direct Shh targets, a critical source of Shh signaling that separates the developing eye field into bilateral eyes becomes mispositioned in ethanol-exposed embryos. We demonstrate that ethanol and loss of *vangl2*, synergistically interact to disrupt polarized cellular protrusions required for proper convergent extension movements.

## Results

### Early embryogenesis is the sensitive time window for *vangl2* mutants

**Fig. 1.**
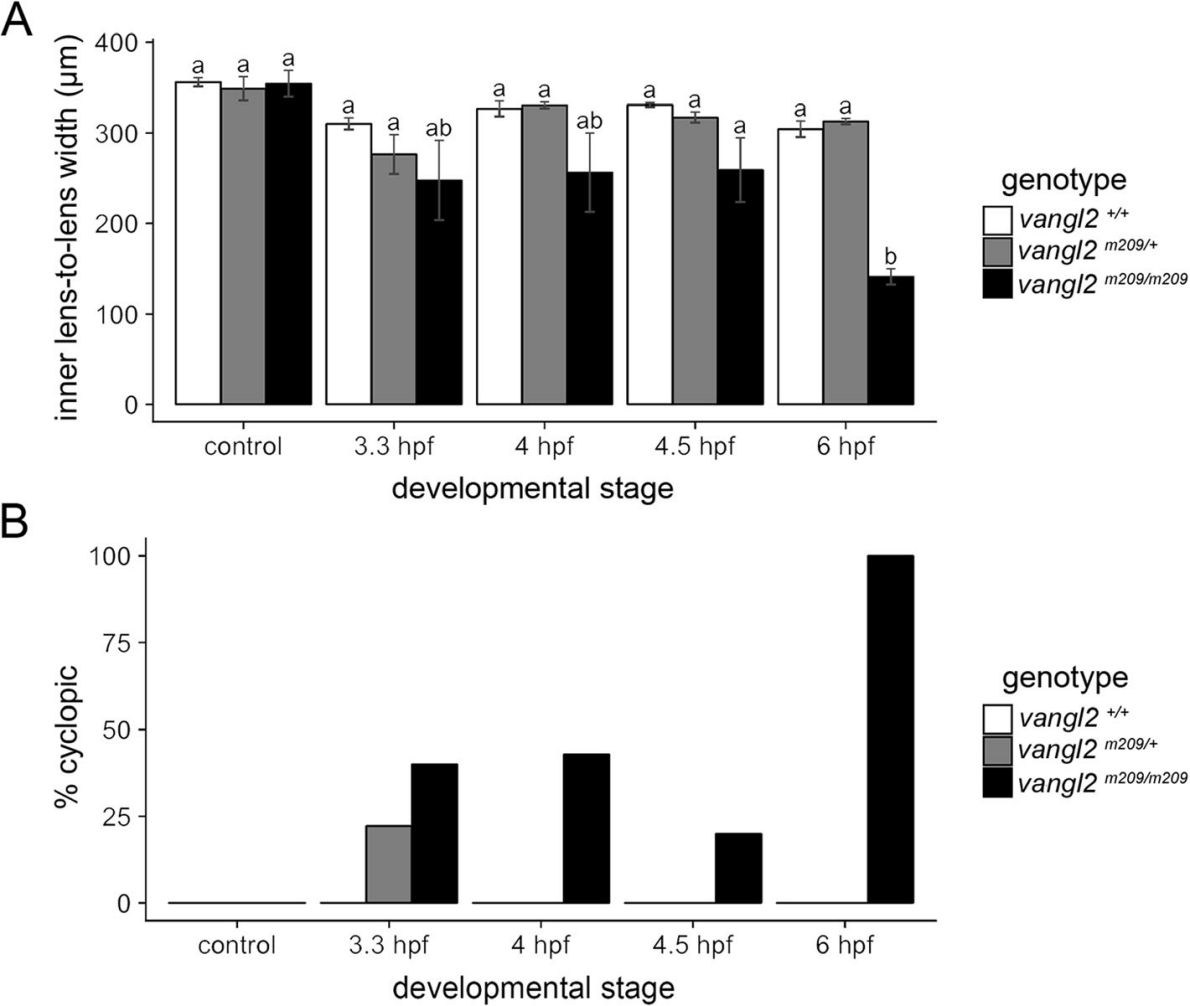
Ethanol interacts with *vangl2* during early embryogenesis. **a** Quantification of the distance between the lenses in embryos treated with 1% ethanol for 24 h, initiating at four different stages [3.3, 4, 4.5, and 6 h post fertilization (hpf)]. Statistical significance of differences between groups is indicated with compact letter display. Groups with different letters are significantly different from one another (p < 0.05; Tukey’s honest test). Sample size and p values provided in Additional file 9: Table S2. **b** Percentage of embryos with complete cyclopia following each treatment. Heterozygous embryos were most sensitive to ethanol when exposed from 3.3 to 27.3 hpf. Mutants were most sensitive from 6 to 30 hpf

To determine the critical time window of ethanol sensitivity for *vangl2* mutants and heterozygotes, we first initiated ethanol treatment at various stages comprising late blastula to early gastrula for 24 h. The inner lens-to-lens width was used as a measure of synophthalmia, and we calculated the occurrence of cyclopia to characterize ethanol-induced teratogenesis. In control conditions, the spacing of the eyes in *vangl2* mutants resembled that of wild-type zebrafish. However, we note that the facial phenotypes in *vangl2* mutants are variable and midline defects can occur even under control conditions [15]. Ethanol-exposed *vangl2* mutants exhibited midline defects ranging in severity from synophthalmia to cyclopia across all time points examined, but these mutants were fully penetrant for cyclopia (100% fused; n=5/5) when ethanol was applied at shield stage (6 h post-fertilization, hpf) at the onset of gastrulation (Fig. 1a-b). Interestingly, heterozygotes only displayed cyclopia when ethanol was applied at a high stage (3.3 hpf) (22% fused; n=4/18), a time when treating wild-type embryos with higher concentrations of ethanol causes similar defects (Fig. 1b) [16, 17]. Thus, heterozygotes and homozygotes are divergent in their time window of greatest ethanol sensitivity. This may be due to a compensatory genetic mechanism in *vangl2* heterozygotes, as zygotic gene expression initiates after high stage (4 hpf) in zebrafish.

### The effect of ethanol on the early zebrafish transcriptome is subtle relative to the developmental age

**Fig. 2.**
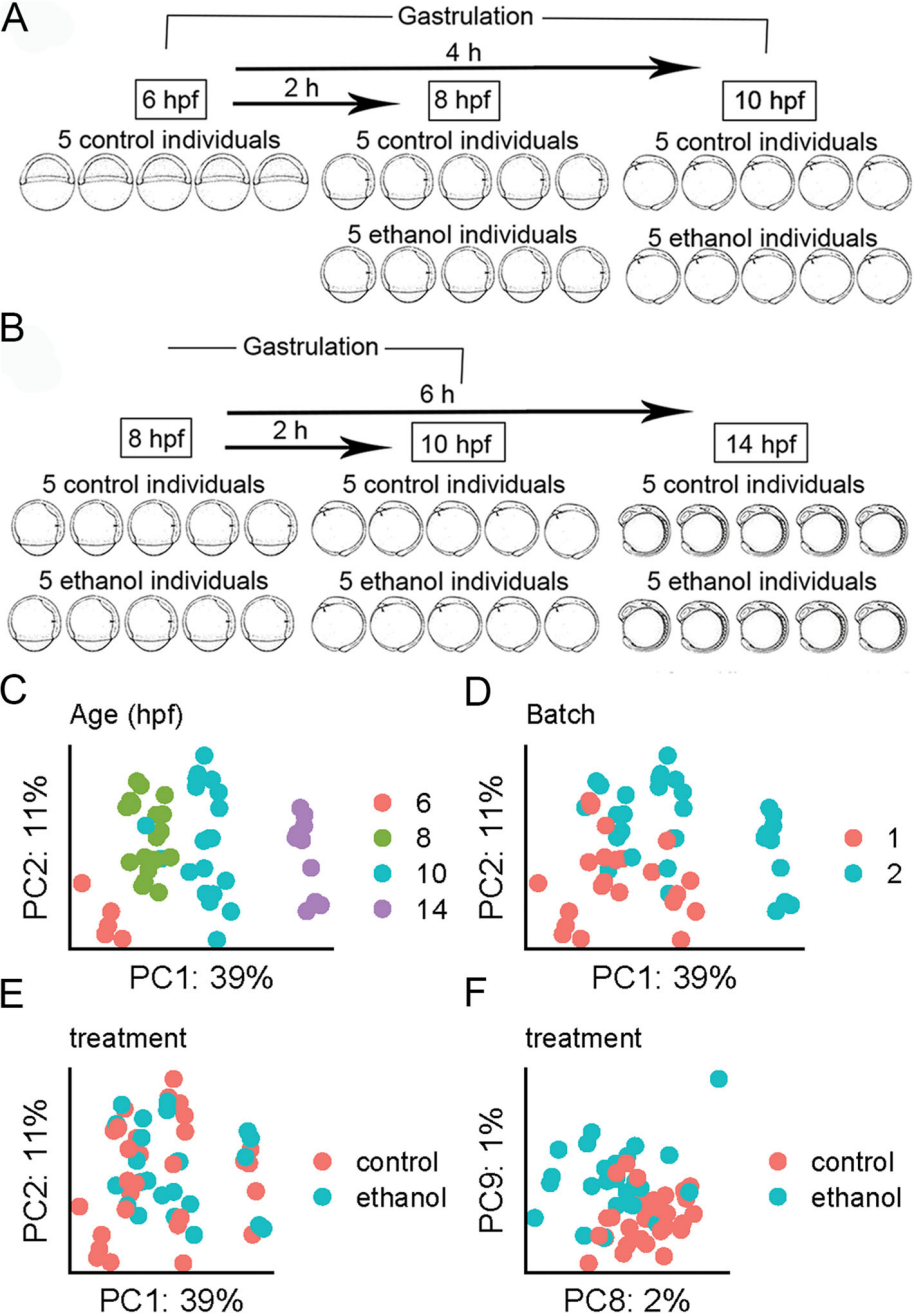
Developmental age is the dominant driver of variation in the dataset. **a** Schematic representation of the RNA-seq experimental design. Wild-type AB embryos were exposed to 1% ethanol at shield stage (6 hpf). Embryos were subsequently collected at 8 and 10 hpf for experiment 1 and **b** 8, 10, and 14 hpf, for experiment 2. **c–f** Principal components analysis (PCA) of the top 25,000 most variable genes. The percentage of variance explained is given on each axis label. **c** *PC1* and *PC2* color coded by developmental age. **d** *PC1* and *PC2* color coded by RNA-seq experiment (batch). **e** *PC1* and *PC2* color coded by ethanol treatment group. **f** *PC8* and *PC9* showing separation of ethanol-treated and control samples

To determine if ethanol caused transcriptional changes that could underlie the interaction with *vangl2*, we designed two RNA-seq experiments that largely overlapped in design (Fig. 2a-b). One possibility for the variability in the phenotypic outcome of ethanol exposure is variable transcriptional response across individuals. Therefore, we performed RNA-seq on single wild-type AB strain embryos to determine if ethanol exposure variably destabilized the transcriptome. Embryos were exposed to 1% ethanol in embryo media (171 mM), which equilibrates to approximately 50 mM tissue concentration [18]. For our RNA-seq experiments, ethanol exposure initiated at 6 hpf, when mutants were the most sensitive. Each sample consisted of an individual zebrafish embryo with five biological replicates per timepoint and treatment. For the first experiment, embryos were collected at mid-gastrulation (8 hpf) and the end of gastrulation (10 hpf) (Fig. 2a). A second experiment was performed to increase power and to include a time when the eye fields have completely separated, 14 hpf (Fig. 2b). The 6 hpf control samples were omitted in the second experiment since they lacked ethanol-treated samples for comparison. The data from both experiments were combined for subsequent analyses, controlling for batch effects.

To assess the effect of age, batch (experiments 1 and 2), and ethanol, on the early zebrafish transcriptome, we performed principal component analysis (PCA). Individuals of similar age clustered tightly along *PC1*, which accounted for 39% of the transcriptional variation observed across the datasets (Fig. 2c). This strong effect of age on the zebrafish transcriptome is in agreement with previous studies [19]. Clustering of samples by age, regardless of ethanol treatment suggested that control and ethanol-treated samples were accurately staged, indicating that ethanol did not delay developmentally regulated transcriptome patterns. There was greater discrimination of 14 hpf samples relative to earlier timepoints (6, 8, and 10 hpf), which is consistent with the greater distinction of this timepoint in terms of developmental time and morphology (Fig. 2c). *PC2* largely captured batch effects between experiments 1 and 2 (Fig. 2d).

The majority of variation between samples did not appear to be due to treatment, with control and ethanol-treated samples randomly interspersed along *PC1* and *PC2* (Fig. 2e). Separation by treatment was observed along *PC8* and *PC9*, which accounts for 3% of the variation in the data (Fig. 2f). Hierarchical clustering of samples based on correlation further corroborated this finding. The 6 and 14 hpf samples showed the greatest dissimilarity, whereas the 8 and 10 hpf samples showed the greatest similarity, irrespective of treatment (Additional file 1: Fig. S1). In summary, the transcriptional effect of exposure to this dose of ethanol on the early zebrafish transcriptome was subtle, while age provided the strongest transcriptional fingerprint.

### Ethanol has effects on transcription that are largely distinct between different developmental timepoints

**Fig. 3.**
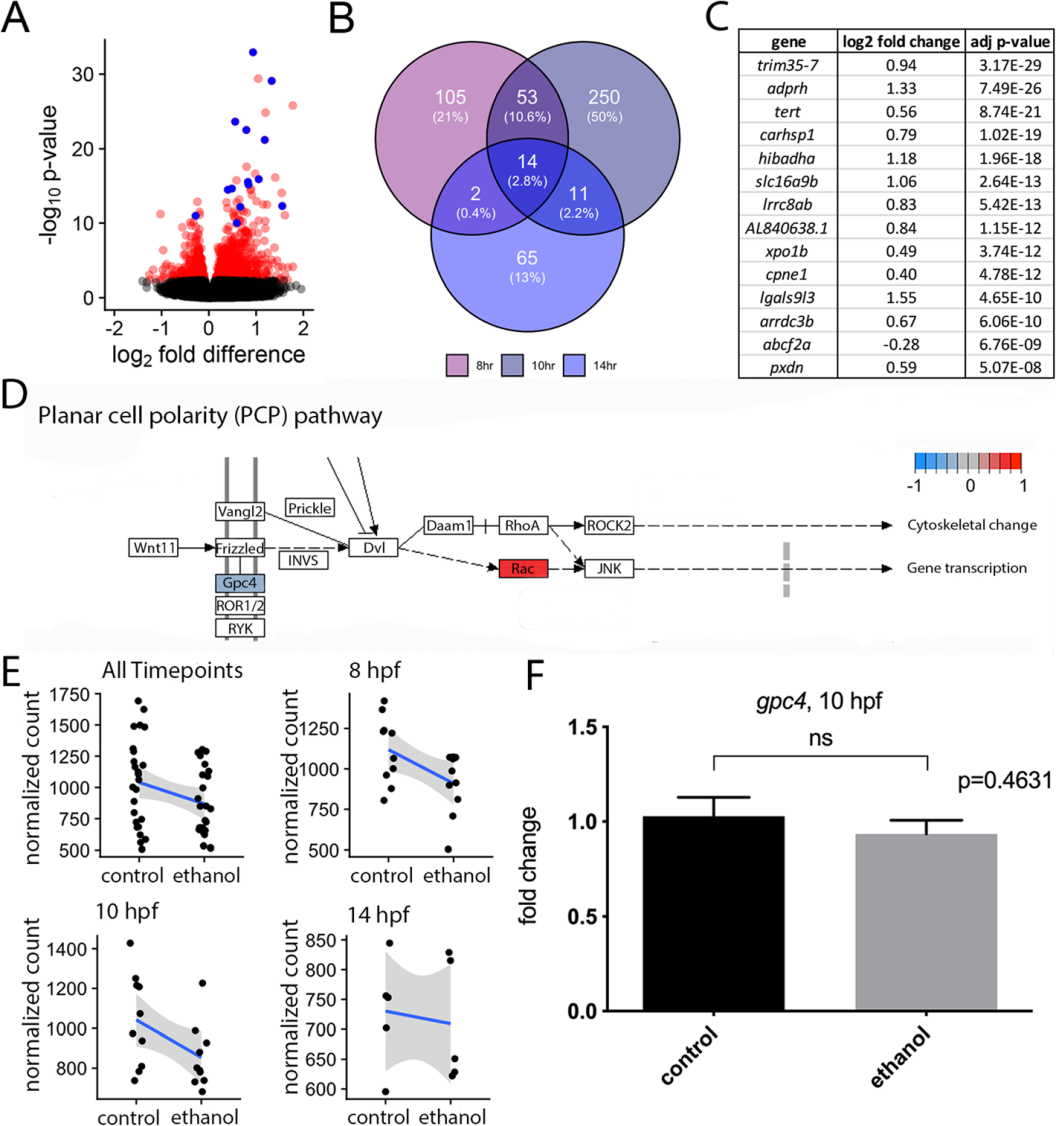
Effects on transcription are largely distinct between developmental time points. **a** Volcano plot showing differential expression due to ethanol treatment across all samples. Significant genes (FDR < 0.1) are indicated in red. Genes that were significant at all time points are indicated in blue. **b** Venn-diagram showing overlap of significant genes between the three individual timepoints. Total number of genes is indicated for each time point and overlapping time points. Percentage of significant genes out of the total number of genes is included in parentheses. **c** Genes that were significant at all timepoints. **d** KEGG pathway schematic illustrating differential expression due to ethanol treatment in the Wnt/PCP pathway. Color coding indicates log_2_ fold differences due to ethanol treatment across all samples. Only pathway members with significant changes were color coded. **e** Normalized read counts indicating expression of *gpc4* across different subsets of the dataset. **f** RT-qPCR of *gpc4* in 10 hpf control and ethanol-treated wild-type embryos. n=7, for each group (p = 0.4631)

Although developmental age was a stronger source of transcriptional variation, we still detected substantial variation in gene expression following ethanol exposure. There were 1414 differentially expressed genes (DEGs), with a false-discovery rate (FDR) less than 0.1 (Fig. 3a; Benjamini–Hochberg procedure). There were more upregulated than downregulated DEGs among ethanol-treated individuals across timepoints (Additional file 2: Fig. S2A-D), and these DEGs were distinct between developmental timepoints (Fig. 3b). In summary, while some genes are generally affected by ethanol across several critical periods, ethanol largely elicits unique responses at distinct developmental stages spanning early embryogenesis.

### Ethanol does not affect the Wnt/PCP pathway at the transcriptional level

While *vangl2* mutants do not typically exhibit cyclopia, cyclopia is observed in double mutants between *vangl2* and other Wnt/PCP pathway members [15, 20]. One potential mechanism for the interaction between ethanol and *vangl2* would be the transcriptional misregulation of other Wnt/PCP pathway members. However, transcription of Wnt/PCP pathway members was largely unaffected by ethanol exposure. No Wnt/PCP pathway members were among the 14 shared DEGs across all timepoints (Fig. 3c). KEGG pathway enrichment analysis further confirmed that ethanol had little effect on the Wnt/PCP pathway at the level of transcription (Fig. 3d). Only two Wnt/PCP pathway members were moderately affected: ethanol exposure moderately decreased expression of the cofactor, *glypican 4* (*gpc4*), (log_2_ fold= −0.237; p value = 0.036) across all timepoints [15, 21] and increased expression of *rac3a* (log_2_ fold = 0.737; p value = 8.89E−06), a member of the Rho family of small GTPases [22]. We plotted the normalized read counts across each time point from the RNA-seq experiment to further investigate the dynamics of the potential alteration in *gpc4* levels. The expression of *gpc4* was modestly reduced in ethanol-treated embryos across the RNA-seq dataset (Fig. 3e). Ethanol exposure consistently downregulated *gpc4* at 8 and 10 hpf, but the magnitude of the downregulation was relatively modest. To statistically compare ethanol-treated and control embryos, expression of *gpc4* at 10 hpf was investigated using real-time quantitative reverse transcription PCR (RT-qPCR). This result demonstrated that *gpc4* was not significantly affected by ethanol exposure (p = 0.4631) (Fig. 3f). Thus, direct transcriptional alteration to the Wnt/PCP pathway is unlikely to explain the ethanol-induced phenotypes in *vangl2* mutants and heterozygotes.

### Modules of co-regulated genes related to ethanol exposure

To determine if ethanol disrupted networks of genes could explain the *vangl2*-ethanol interaction, we next performed Weighted Gene Co-expression Network Analysis (WGCNA) [23]. This unsupervised network analysis identifies groups of genes, termed “modules,” based on correlated expression patterns across the samples. Modules are summarized by the first principal component for the expression estimates of the included genes, termed the “module eigengene,” which can be correlated with sample traits to identify biological significance. The cluster dendrogram generated in this analysis illustrates the presence of highly distinct and clustered modules (Additional file 3: Fig. S3A). The merging of similar modules produced eleven total modules (Additional file 3: Fig. S3B). Consistent with our PCA analysis, the module eigengenes were primarily correlated with time. We used these modules to further validate our ethanol exposure regimen.

Previous work has shown that ethanol delays development in a dose-dependent manner at concentrations equal to or greater than 1.5% ethanol [24]. For the experiments herein, ethanol-treated samples were morphologically stage-matched to control samples to exclude differences due to developmental age or delay. To confirm that the ethanol samples were indeed age-matched to the control samples at the level of transcription, we compared expression patterns of developmentally regulated genes between the ethanol and control samples from each timepoint. For developmentally regulated genes, we used the gene with the highest module membership, the “hub gene,” from each of the WGCNA modules that was associated with time (p < 0.05) (Additional file 4: Fig. S4). Consistent with the results from the PCA, the slope of the expression levels for each gene were similar in control and ethanol-treated samples across age, most clearly demonstrated in the magenta4, thistle1, and blueviolet modules (Additional file 4: Fig. S4B-C, and G). Interestingly, we find a time-specific difference in the expression of hub genes in the honeydew1, greenyellow, and saddlebrown modules at 10 hpf (Additional file 4: Fig. S4F, H, and K). Together, these data indicate samples were accurately age-matched and the observed changes were biologically relevant.

Two modules (mediumpurple4 and darkolivegreen4) correlated with ethanol treatment (Additional file 3: Fig. S3B-C). The purple and green modules positively and negatively correlated with ethanol treatment, respectively (Additional file 3: Fig. S3D). A significant differentially expressed gene from the green (Additional file 3: Fig. S3E) and purple module (Additional file 3: Fig. S3F) were selected for independent validation on biological replicate samples derived from the same wild-type zebrafish line using RT-qPCR. These results indicate that our RNA-seq faithfully represents transcript levels. The green module is only weakly downregulated and is enriched for genes encoding zinc finger (ZnF) proteins, which included *znf1015* (Additional file 3: Fig. S3G). This module is almost entirely composed of genes on chromosome 4 that have been shown to be co-regulated during these stages of development [25, 26]. The purple module revealed GO enrichment of transmembrane transporters, which included *slc16a9a* (Additional file 3: Fig. S3H). As an upregulated module enriched in transporters, these genes are likely to represent the physiological response to ethanol. Thus, while we did identify two modules associated with ethanol (Additional file 5: Table S1), the module membership did not provide clues to the interaction between ethanol and *vangl2*.

### Cyclopamine is predicted to mimic the effects of ethanol

One challenge in RNA-seq analyses is inferring the mechanism underlying a diseased or environmentally perturbed state from a large set of differentially expressed genes. Individual functional analyses of significant gene-ethanol interactions are time-consuming and thus inefficient. To circumvent this problem, we adopted a bioinformatics approach, utilizing the Library of Integrated Network-Based Cellular Signatures (LINCS L1000) toolkit [27]. This transcriptomic dataset includes gene expression data from nine human cancer cell lines exposed to thousands of small molecule drugs [27]. We queried the top 100 and 150 upregulated and downregulated genes induced by ethanol exposure against the LINCS L1000 dataset using the clue.io platform (https://clue.io). The query generated a list of small molecules predicted to have a positive or negative correlation to the input signature (i.e., small molecules predicted to either mimic or antagonize the transcriptional effects of ethanol). We were particularly interested in those chemicals with a positive correlation to ethanol as they would give insight into the mechanism of ethanol teratogenicity and highlight potential co-factors that exacerbate ethanol teratogenicity.

Interestingly, cyclopamine, a hallmark Shh pathway inhibitor that inhibits the core Shh pathway protein Smoothened (Smo), was predicted to positively correlate with the ethanol signature. The Shh pathway is critical for midfacial development and mice deficient in *Sonic Hedgehog* (*Shh*) exhibit severe brain and face malformations, including holoprosencephaly and a single-medial eye (cyclopia) [28]. In zebrafish, reduction of *shh* or null mutations in *smo* similarly results in severe loss of craniofacial midline structures of the anterior neurocranium [29]. Animal models have demonstrated ethanol is an environmental risk factor for holoprosencephaly, resulting in a characteristic set of midfacial defects, a hypomorphic forebrain, and in severe cases, cyclopia [16, 17, 30–33]. Thus, attenuation of the Shh pathway could mechanistically explain the *vangl2*-ethanol interaction.

### Ethanol indirectly attenuates Shh signaling

**Fig. 4.**
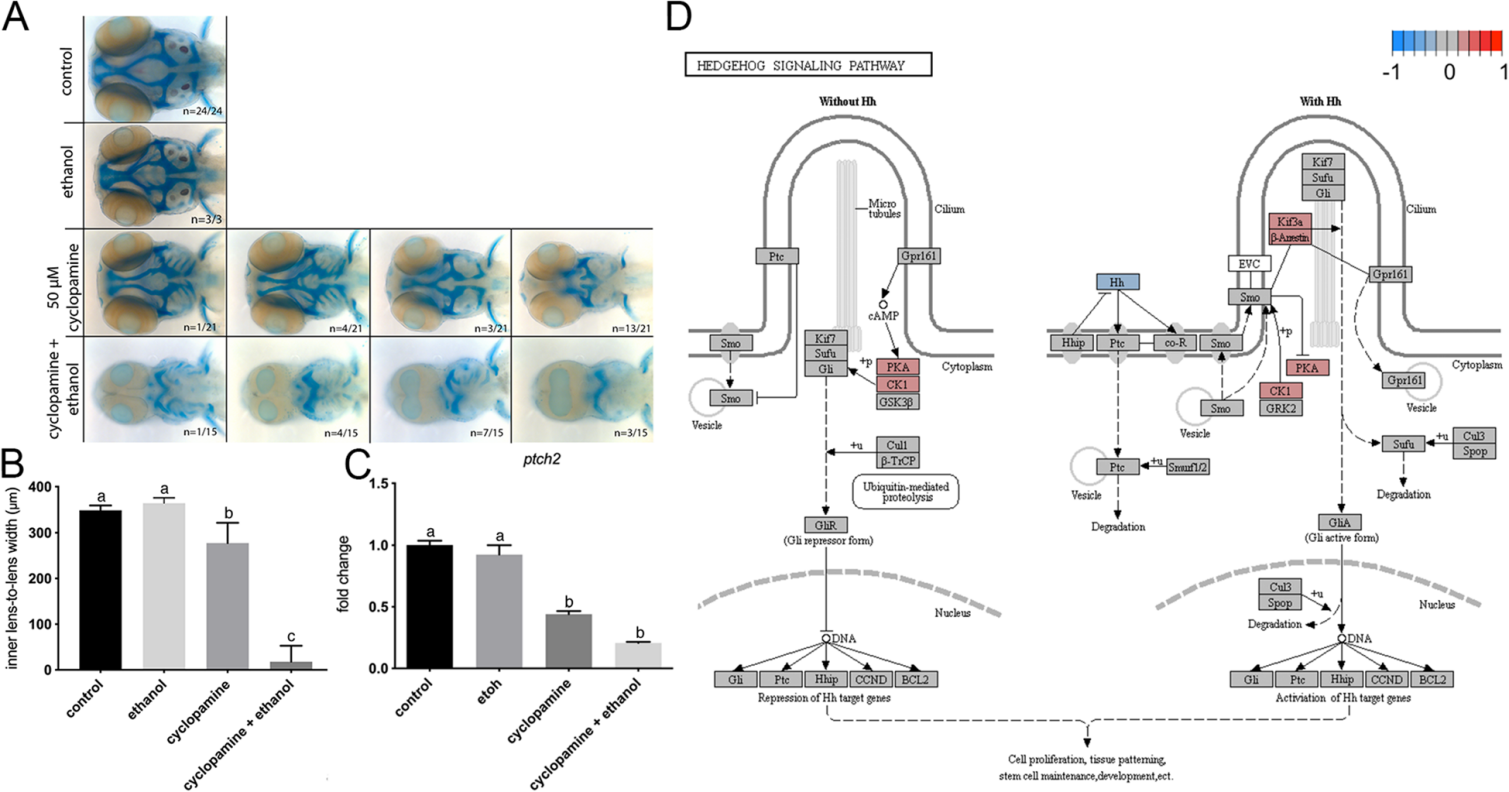
Ethanol indirectly attenuates Shh signaling. **a** Alcian blue and Alizarin red whole mount staining of 4 dpf control, cyclopamine-treated (50 μM), and ethanol- and cyclopamine-treated (1% ethanol plus 50 μM cyclopamine) wild-type embryos from 6 hpf to 4 dpf. Horizontal panels represent the spectrum of phenotypes observed for the treatment groups. Dorsal view, anterior to the left. **b** Quantification of the effect on inner lens-to-lens width. Both cyclopamine alone and cyclopamine and ethanol were significantly different from controls (p < 0.0001; Tukey’s honest test). Sample size and p values provided in Additional file 9: Table S2. **c** RT-qPCR of *ptch2* in 10 hpf control and ethanol- and/or cyclopamine-treated wild-type embryos. n = 4, for all groups (p < 0.05; Tukey’s honest test). P values provided in Additional file 9: Table S2. **d** KEGG pathway schematic illustrating differential expression due to ethanol treatment in the sonic hedgehog (Shh) signaling pathway. Color coding indicates log_2_ fold differences due to ethanol treatment across all samples

To investigate the interaction of cyclopamine and ethanol, we first exposed wild-type zebrafish embryos to cyclopamine (50 μM) at shield stage (6 hpf) for 24 h, mimicking the ethanol exposure window for the *vangl2* mutants. Embryos were fixed at 4 dpf and the cartilage and bone were stained with Alcian blue and Alizarin red, respectively. We observed a range of midfacial defects with the most severe phenotype being a complete loss of the anterior neurocranium and reduced spacing between the eyes (Fig. 4a). Since cyclopamine was predicted to mimic the effects of ethanol, we next combined this low dose of cyclopamine with ethanol. Strikingly, all embryos presented with synophthalmia or cyclopia and significant reductions and defects of the cartilages of the neuro- and viscerocranium. To quantify this combinatorial effect, we measured the inner lens-to-lens width and observed a significant reduction in co-exposed embryos relative to those exposed to either ethanol or cyclopamine alone (p < 0.0001), suggesting a strong synergistic interaction (Fig. 4b).

In chick, ethanol exposure during somitogenesis has been proposed to suppress Shh signaling and induce apoptosis in cranial neural crest cells that make up the craniofacial skeleton [34]. However, work in zebrafish shows only a modest increase in cell death within the eye field at a much higher dose of 2% ethanol [35]. To ensure that cells in the eye field are not simply undergoing apoptosis, we performed a TUNEL cell death assay in ethanol-treated *vangl2* mutants at 11 hpf, prior to optic vesicle evagination. As expected, we failed to detect an increase in apoptotic cells within the eye field in ethanol-treated *vangl2* mutants or their siblings (Additional file 6: Fig. S5). Thus, Shh-mediated pro-survival signals do not appear to be significantly disrupted by ethanol.

To test for a direct effect of ethanol on Shh signaling, we quantified the relative expression of *patched 2* (*ptch2*), a canonical read-out of Shh pathway activity, at bud stage (10 hpf). Ethanol exposure had no effect on the expression levels of *ptch2* (p = 0.9966), consistent with RNA-seq results (Fig. 4c). While cyclopamine significantly reduced Shh signaling (p = 0.0006), ethanol did not further reduce *ptch2* levels significantly (p = 0.1115). KEGG analysis from the RNA-seq confirmed that ethanol does not affect the Shh pathway as a whole, nor does it reduce the levels of direct targets of Shh signaling (i.e., *gli*, *ptch*) (Fig. 4d). Thus, at this dose, ethanol does not appear to directly attenuate Shh signaling.

### Ethanol disrupts convergent extension

The ethanol-induced *vangl2* mutant phenotype closely mirrors those in compound mutants between *vangl2* and other Wnt/PCP pathway members [15, 20]. These double mutants display a further reduction in convergent extension, as evidenced by a shorter and broader body axis [15]. Based on these data, we hypothesized that ethanol disrupts convergent extension, which would mislocalize the Shh signal and result in eye defects.

**Fig. 5.**
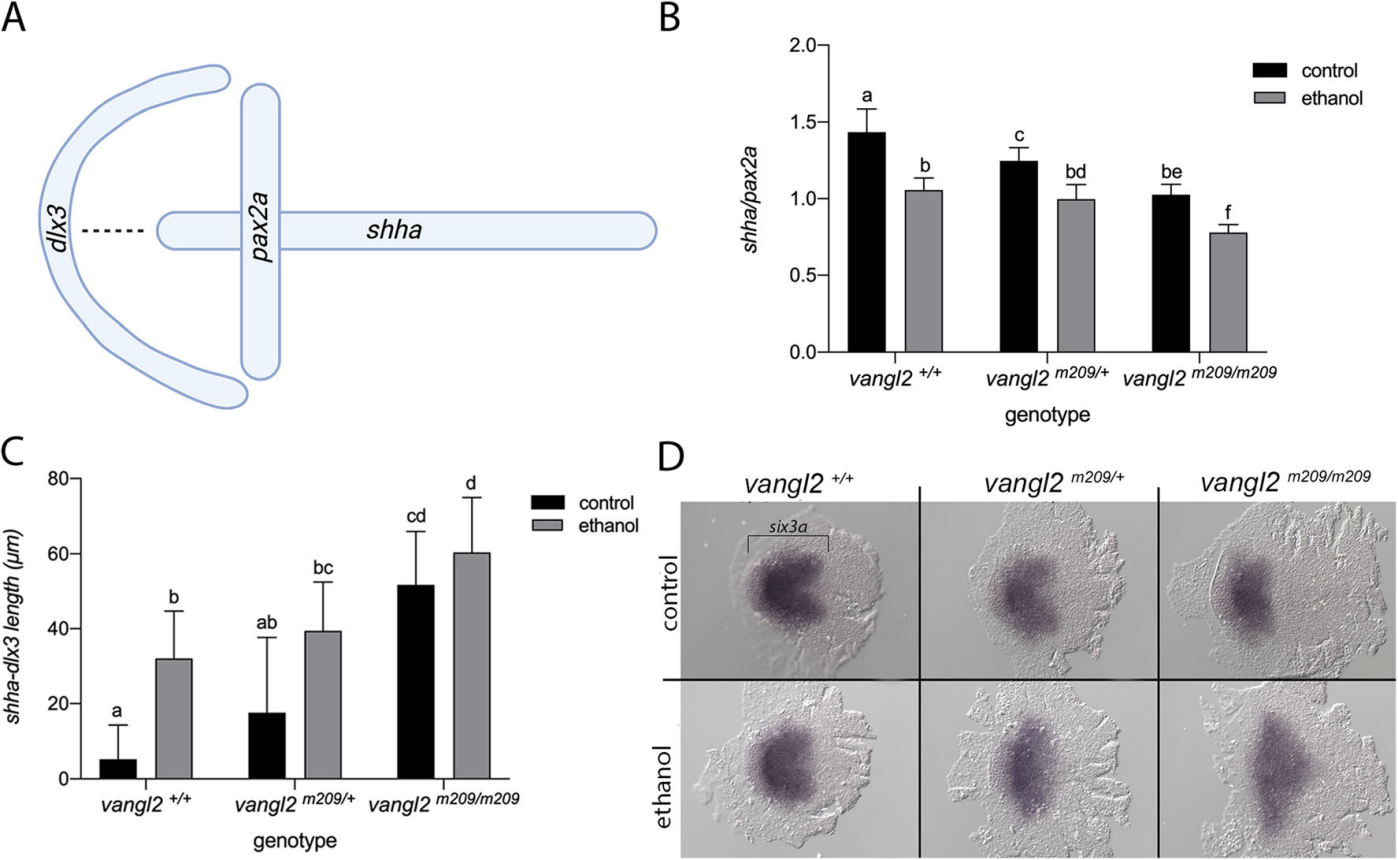
Ethanol disrupts convergent extension. **a** Schematic of the in situ expression domains of *shha* (midline), *pax2a* (midbrain-hindbrain boundary), and *dlx3* (anterior neural plate) at 10 hpf. The dotted line indicates the *shha-dlx3* length measured in panel C. Dorsal view, anterior to the left. Created with BioRender.com. **b** Quantification for the normalized *shha*/*pax2a* expression domains (p < 0.05; Tukey’s honest test). Sample size and p values provided in Additional file 9: Table S2. **c** Quantification of the distance between *shha* and *dlx3*, as shown by the dotted line in panel A (p < 0.05; Tukey’s honest test). Sample size and p values provided in Additional file 9: Table S2. **d** Expression pattern of *six3a* via whole mount in situ hybridization, in control and ethanol-treated *vangl2* embryos at 11 hpf

We performed in situ hybridization on control and ethanol-treated *vangl2* embryos to examine convergent extension and to test the hypothesis that the Shh expression domain is mislocalized in ethanol-exposed *vangl2* mutants. We analyzed the expression of *shha* in the axial mesoderm and *paired box 2a* (*pax2a*) in the midbrain-hindbrain boundary at bud stage (10 hpf) (Fig. 5a) [36]. Ethanol was initiated at a high stage (3.3 hpf). We chose this timepoint because it is when heterozygotes are most sensitive to ethanol-induced synophthalmia. Because *vangl2* mutants have convergent extension defects and display synophthalmia, we reasoned that effects may be more apparent in heterozygotes. We observed a gene and ethanol-dose-dependent reduction in the length of the *shha* expression domain (extension) and an increase in the width of the *pax2a* expression domain (convergence).

To quantify the effect of ethanol on convergent extension, we plotted the normalized expression values of *shha*/*pax2a* (Fig. 5b). Post hoc analyses (Tukey’s) revealed significant differences between control embryos across *vangl2* genotypes, indicating that *vangl2* gene dosage affects convergent extension. Similarly, we found significant differences between ethanol-treated mutants and their heterozygous (p = 0.0037) and wild-type siblings (p = 0.0052). This observation confirms that loss of *vangl2* results in reduced convergent extension movements, as evidenced by their short body axis. This phenotype was exacerbated with ethanol treatment, resulting in reduced convergent extension for *vangl2* heterozygotes and homozygotes, compared to their untreated counterparts. While there was a trend, we did not observe a difference in convergent extension between ethanol-treated *vangl2* heterozygotes and their wild-type siblings in their *shha*/*pax2a* ratio (p = 0.9554). However, synophthalmia occurred in ethanol-treated heterozygotes but not wild-type embryos, demonstrating that the nonsignificant reduction in convergent extension in the heterozygotes can have a phenotypic consequence.

Separation of the eye field into two eye primordia is accomplished via Shh signaling to the anterior neural plate. We measured the distance between *shha* and *dlx3*, a marker of the anterior neural plate (Fig. 5a and c), to determine if ethanol alters the source and target of Shh. Here again, we find a combinatorial effect of ethanol and *vangl2* gene dosage, with ethanol-exposed mutants having the greatest distance between the *shha* and *dlx3* expression domains (Fig. 5c). Ethanol exposure has a significant effect on this distance in wild-type embryos (p = 0.003). The distance is slightly, albeit non-significantly, elevated in ethanol-treated mutants, compared to unexposed mutants (p = 0.7133). Given that *vangl2* mutants are predisposed to cyclopia, this slight increase may again, have phenotypic relevance. These results are consistent with a model in which the interaction between ethanol and *vangl2* is due to an indirect effect of ethanol on the Shh pathway via combined disruption of convergent extension cell movements.

### Ethanol alters *six3* and *rx3* expression in the eye field

Transcription factors involved in the specification of the eye field have also been implicated in the mechanism of eye field separation. The expression of three of these transcription factors, *six3*, *rx3*, and *rx1* is altered by ethanol exposure [35]. We examined the effect of ethanol on *six3a* and *rx3* in ethanol-treated *vangl2* mutants at the initiation of optic vesicle evagination. In situ hybridization of *six3a* in 11 hpf embryos shows a heart-shaped expression pattern in the prospective forebrain. The caudal indentation marks the splitting of the eye field into bilateral domains (Fig. 5d). This expression pattern becomes more diffuse with loss of *vangl2*. In untreated homozygous mutants, we observed a shortening along the anterior-posterior (AP) axis and a broadening along the mediolateral axis at 11 hpf, consistent with the convergent extension defect. This expression pattern was further exacerbated in ethanol-treated mutants with complete loss of the caudal indentation. We observed a similar effect of genotype and ethanol on *rx3* expression at mid-evagination (12 hpf) (Additional file 7: Fig. S6). At this stage, *rx3* is localized to the prospective forebrain and retina [37]. Ethanol-treated *vangl2* homozygous mutants exhibit a compressed expression domain, clearly displaying a reduction in convergent extension. Our expression analyses suggest the eye field is specified but mislocalized and fails to separate into bilateral domains, likely due to defects in mesodermal migration. However, we cannot rule out a specific transcriptional effect of ethanol on a small cell population that receives Shh signaling, and scRNA-seq would prove useful to identify such interactions.

### Mutation in *gpc4* enhances cyclopia in a dose-dependent manner

Our data support a model in which ethanol interacts with *vangl2* via a combinatorial disruption of convergent extension. If ethanol disrupts convergent extension, which leads to interactions with *vangl2*, then further genetic disruption to convergent extension should exacerbate the ethanol-*vangl2* phenotype. Embryos deficient in *gpc4* similarly have a defect in convergent extension as evidenced by their shortened, broadened body axis [15]. Previous work in zebrafish has shown a functional interaction between *vangl2* and *gpc4*, where *vangl2;gpc4* double mutants were invariably cyclopic [15]. We conducted additional functional analyses to further examine the relationship between these two genes in the context of ethanol exposure. Consistent with Marlow et al., double mutants were fully penetrant for cyclopia with or without ethanol. While 1% ethanol exposure altered the facial morphology of *gpc4* mutants, it did not cause cyclopia (Additional file 8: Fig. S7A). However, ethanol concentrations greater than 1%, which did not cause cyclopia in wild-type siblings, resulted in cyclopia in *gpc4* mutants (Additional file 8: Fig. S7B) [15]. Additionally, embryos carrying 3 mutant alleles (either *vangl2*^*m209/m209*^*;gpc4*^*fr6/+*^ or *vangl2*^*m209/+*^*;gpc4*^*fr6/fr6*^) were greatly sensitized to cyclopia when exposed to 1% ethanol (Additional file 8: Fig. S7C). Collectively, these data indicate that ethanol disrupts convergent extension, and in sensitized genotypes, mispositions a source of Shh that is essential for the separation of the eye field into bilateral primordia.

### Blebbistatin phenocopies the ethanol-induced defect in *vangl2* mutants

**Fig. 6.**
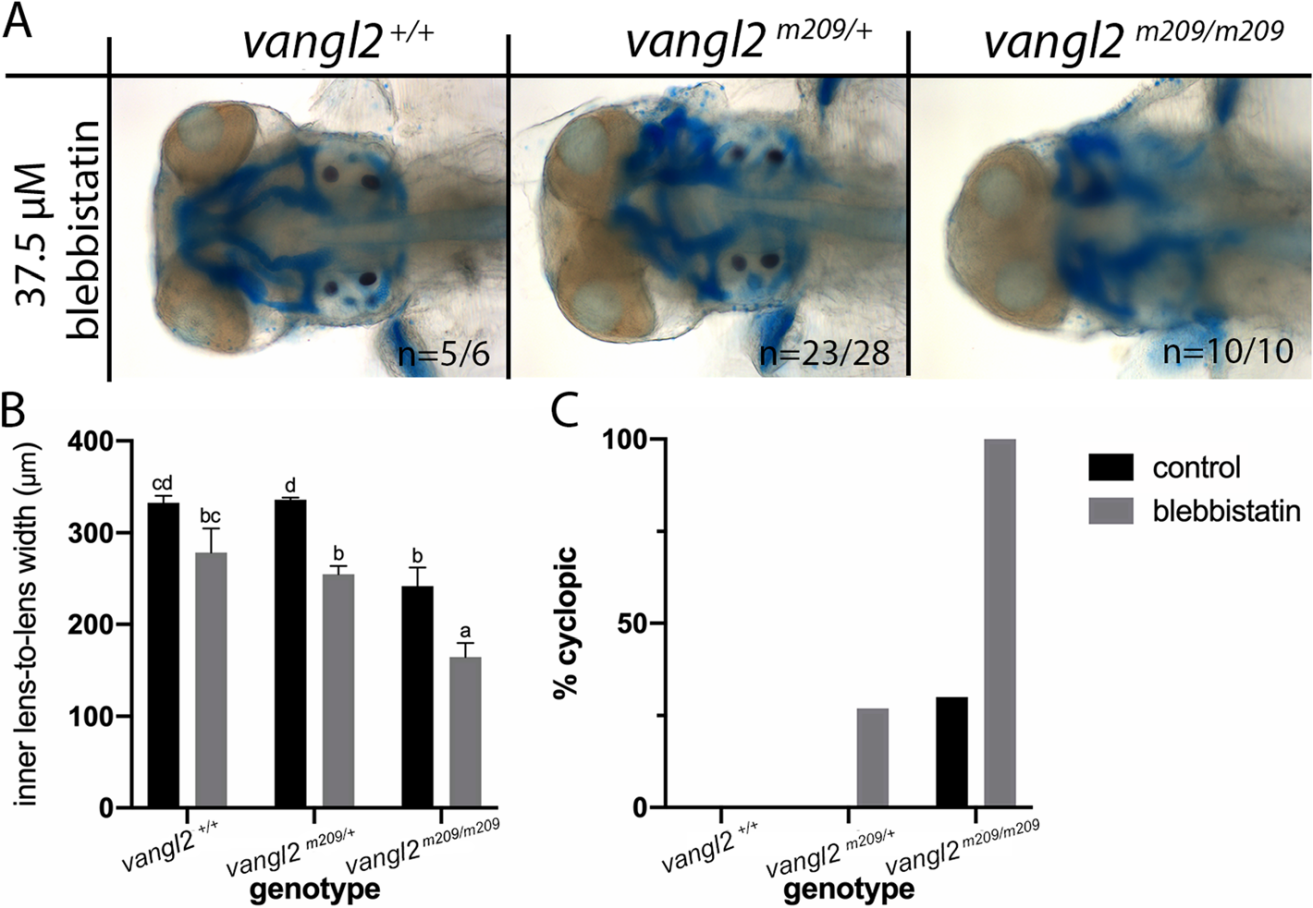
Blebbistatin phenocopies ethanol treatment in *vangl2* mutants. **a** Alcian blue and Alizarin red whole-mount staining of 4 dpf *vangl2* embryos treated with blebbistatin (37.5μM, 6–10 hpf). Dorsal view, anterior to the left. **b** Quantification of the inner lens-to-lens width of control and blebbistatin-treated embryos (p < 0.05; Tukey’s honest test). Sample size and p values provided in Additional file 9: Table S2. **c** Percentage of embryos exhibiting cyclopia

If ethanol is having an effect on convergent extension, a known inhibitor of convergent extension should similarly interact with *vangl2*. We extended our analyses to test the effect of blebbistatin, a myosin II inhibitor that disrupts cell movements and reduces axis elongation, in *vangl2* mutants [38]. Blebbistatin treatment during gastrulation (6–10 hpf) induced synophthalmia in *vangl2* heterozygotes and homozygotes (Fig. 6a). We then quantified this interaction by measuring the inner lens-to-lens width and found a significant reduction in blebbistatin-treated *vangl2* homozygotes (Fig. 6b). Furthermore, 26.92% and 100% of blebbistatin-treated heterozygotes and homozygotes, respectively, were cyclopic (Fig. 6c). These data suggest ethanol further inhibits convergent extension in the sensitized background, *vangl2*, which not only disrupts their ability to elongate their body axis, but also inhibits the splitting of the eye field.

### Ethanol affects *vangl2* filopodia organization on migrating cells

Based on our data, cell dynamics underlying convergent extension are likely involved in the *vangl2*-ethanol interaction. During gastrulation, mesodermal cells adopt a bipolar shape and orient their actin-based cytoskeletal projections on their leading and trailing edge, with respect to the notochord, allowing for proper convergent extension [39]. In *vangl2* mutant embryos, these gastrula cells fail to elongate and align with the primary axis [40]. Furthermore, *vangl2* mutant ectodermal cells possess a greater number of filopodial protrusions than their wild-type siblings and display projections in an unpolarized manner [41].

**Fig. 7.**
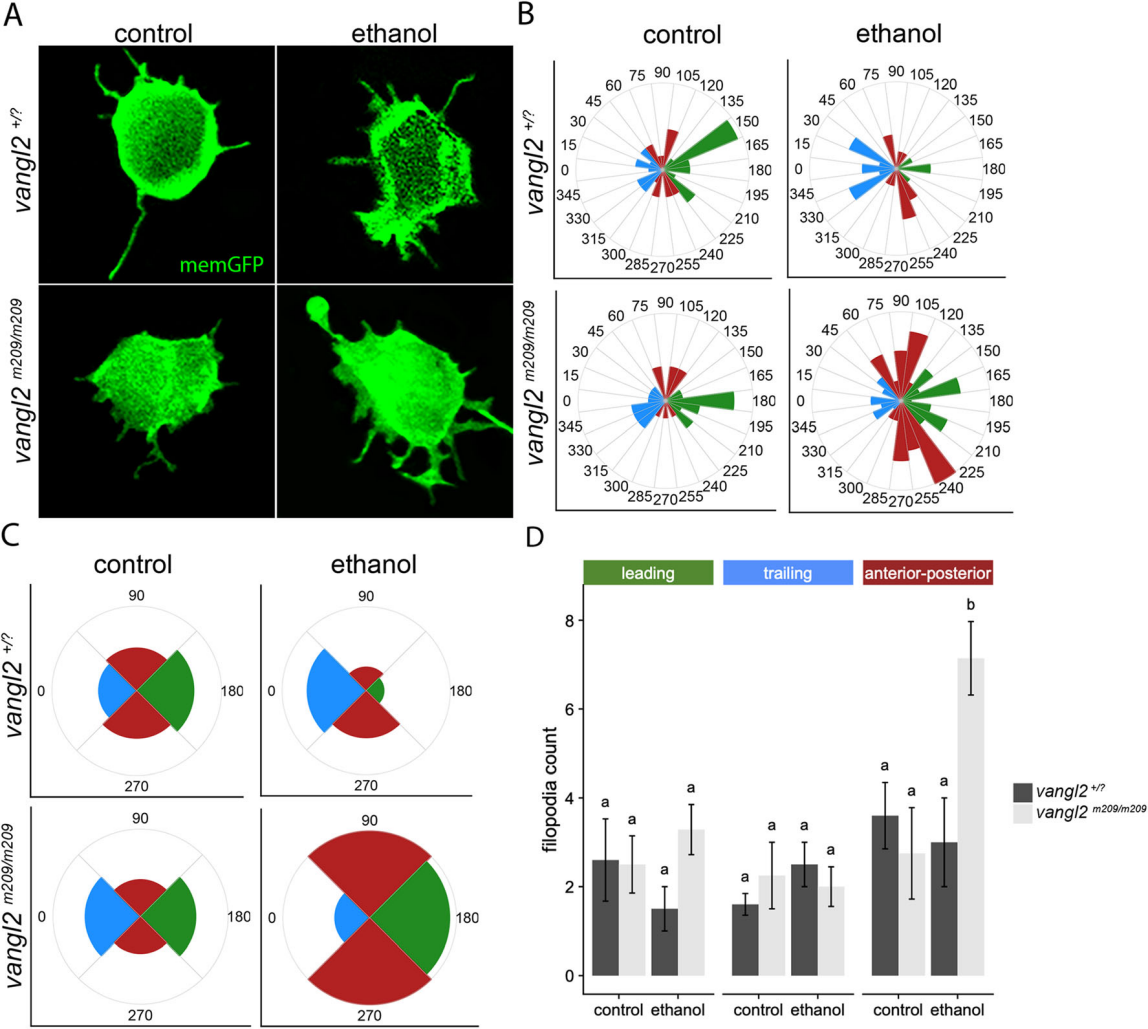
Ethanol disrupts the polarity of membrane protrusions in *vangl2* mutants. **a** Confocal images of mesodermal cells expressing memGFP at 10 hpf. Ethanol treatment from 6–10 hpf. **b–c** Rose diagram of filopodial projections representing length and distribution around the cells, using **b** 24 bins, divided into 15^°^ angles or **c** 4 bins, divided into 90^°^ angles. Horizontal plane (0^°^) represents the mediolateral axis; vertical plane (90^°^) represents the anterior-posterior axis. **d** Quantification of the number of filopodia. n=6, for each group (p < 0.05; Tukey’s honest test). P values provided in Additional file 9: Table S2

To determine whether ethanol may be exacerbating this phenotype, we analyzed filopodia protrusion length and orientation around the circumference of migrating live mesodermal cells. We injected memGFP mRNA into a single blastomere of *vangl2* embryos at the 8–32 cell stage, to obtain mosaic expression. We then treated a subset of the injected embryos with 1% ethanol from 6 to 10 hpf. Homozygous mutants were identified based on their reduced body axis elongation at 10 hpf. Cells were imaged at 40–60^°^ from the primary axis. Spike-like filopodia were highly unpolarized in ethanol-treated *vangl2* mutants compared to their wild-type and heterozygous siblings (Fig. 7a). We quantified the polarity and number of filopodia for each cell, with respect to the head (90^o^), the primary axis/notochord (180^o^), and the tail (270^°^) (Fig. 7b). Rose diagrams suggest ethanol-treated *vangl2* mutants have more un-polarized projections on the anterior/posterior edge (red) as opposed to the leading (green) or trailing (blue) edge. We next reduced the number of bins in the rose diagrams to 90^°^ and noticed a clear over-representation of filopodia in *vangl2* mutants on their anterior/posterior edge (Fig. 7c). Tukey’s post hoc analyses confirmed ethanol-treated *vangl2* mutants had significantly more filopodia on their anterior/posterior edge compared to other axes and their wild-type or heterozygous siblings (Fig. 7d). These data confirm that ethanol exposure is associated with an increase in the number of filopodial projections oriented in the wrong direction, or perpendicular to the path of dorsal migration, in ethanol-treated *vangl2* mutants.

## Discussion

### Molecular staging for the early zebrafish embryo

Despite their importance, the nature and mechanisms of gene-environment interactions are largely unknown. Here, we performed single embryo bioinformatic analyses to shed light upon the mechanism behind gene-environment interactions. A growing number of researchers are using the zebrafish for toxicological and gene-environment studies. One potential confounding variable in these types of studies is developmental delay. Our WGCNA analyses are rich in gene modules that similarly vary over developmental time, providing a way to molecularly stage embryos. Thus, our results will provide important resources to ensure the rigor and reproducibility of these analyses.

### The effect of ethanol on the transcriptome is subtle

Our hierarchical clustering analysis demonstrates that the effect of ethanol on the transcriptome is minor. Within any given time point, ethanol-exposed and unexposed embryos intermingled. PCA results support this, demonstrating that only 3% of the variance in the transcriptomic data is attributable to ethanol exposure. In sum, we found 1414 differentially regulated genes across all time points with the majority of these genes being upregulated. Most differentially expressed genes were specific to a single time point. At any given time point, there were between 92 and 328 differentially expressed genes. Thus, exposure to a dose of ethanol that does not disrupt facial development results in subtle and dynamic alterations to the transcriptome.

Our findings identified dramatically fewer differentially expressed genes than those observed in transcriptomic analyses of ethanol-exposed chicken and mouse embryos. In chicken 3422 differentially regulated genes were observed after 6 h of ethanol treatment and the preponderance (1924 genes) were downregulated [42]. In a mouse microarray, 2906 differentially expressed genes were identified 3 h after ethanol injection [43]. Species-specific developmental trajectories or the tissue analyzed, cranial neural fold (chicken), headfold (mouse), versus whole embryo (our study) may account for some of these differences. However, it is important to note that the chicken and mouse strains analyzed are highly sensitive to the dose of ethanol used while our zebrafish AB strain is not (without the addition of a sensitizing mutation). Thus, the chicken and mouse data likely represent transcriptomic changes representative of pathogenesis. Our zebrafish data reflect a direct effect of ethanol, which may or may not be pathogenic depending upon the genetic and environmental milieu.

WGCNA analysis identified two modules (mediumpurple4 and darkolivegreen4) of genes that are coordinately altered by ethanol exposure. The vast majority of genes that negatively correlate with ethanol exposure (membership in darkolivegreen4) encode zinc finger proteins (ZnF) located on chromosome 4. Ethanol has similarly been shown to downregulate *Zinc finger protein, subfamily 1A*, 4 in mouse fetuses exposed to ethanol during early development [44]. However, the functional significance of these changes in ZnF proteins remains unknown and they often have diverse binding affinities and functions. The long arm of zebrafish chromosome 4 (*Chr4q*) is typically heterochromatic (condensed) and lacking in protein-coding genes [45]. However, it is not late replicating until the end of gastrulation or bud stage (10 hpf) [26]. Previous work in zebrafish has found ZnF proteins on chromosome 4 to undergo robust expression from the initiation of zygotic transcription until mid-gastrula stage [25]. Since many chromosome 4 genes are downregulated across ethanol-treated individuals during gastrulation, ethanol may interfere with replication timing, blocking the early-to-late replication switch, or chromatin remodeling. Based on these findings, the differentially expressed genes may follow the disrupted expression of ZnF proteins due to chromatin remodeling.

### Ethanol interacts with *vangl2* through the indirect effects on the Shh pathway

Because Vangl2 is a core member of the Wnt/PCP pathway, a simple explanation for ethanol-sensitivity in *vangl2* mutants would be that ethanol transcriptionally dysregulates the Wnt/PCP pathway. However, our RNA-seq results indicated that this is not the case. The expression of *gpc4* was the only member of the Wnt/PCP pathway that was downregulated in our RNA-seq dataset. Cyclopia is observed in *vangl2;gpc4* double mutants [15], and we show that heterozygosity for *gpc4* further sensitized *vangl2* mutants to ethanol. However, the downregulation of *gpc4* was quite modest and unlikely to have any substantial impact on the phenotype of ethanol-exposed *vangl2* mutant.

We used an unbiased approach, the LINCS L1000 dataset to identify pathways that might be disrupted by ethanol and generate cyclopia in *vangl2* mutants. One of the top matches to the effects of ethanol was cyclopamine, a Hh pathway inhibitor. Shh signaling is critically important for the development of the midface across species [28, 46–48]. In zebrafish, *shha* and *smo* mutants have cyclopia because Shh signaling to the diencephalon is critical for the separation of the eye fields [49, 50]. Furthermore, cyclopia observed in Wnt/PCP pathway mutants is due to the mispositioning of this source of Shh [16, 20]. Thus, disruption of the Shh pathway could mechanistically explain the ethanol sensitivity of *vangl2* mutants.

The Shh pathway has been implicated in ethanol teratogenesis. However, there has been much controversy regarding whether this interaction is direct or not [8]. As predicted by LINCS, we find a synergistic interaction between ethanol and the Hh pathway inhibitor cyclopamine. However, both our RNA-seq and qPCR data demonstrate that, at this timing and dose, ethanol does not alter the expression of direct readouts of Shh activity. Such an alteration would be expected if ethanol interacted directly with core components of the pathway. As supported by our in situ analysis, we conclude that ethanol indirectly affects the Shh pathway by mislocalizing the source of the ligand. While there is no global disruption to Shh signaling, it remains possible that there are cell-type-specific effects to the pathway. Such effects could be difficult to identify in our whole embryo approach. Single-cell RNA-seq would be useful in identifying cell type-specific effects of ethanol on transcription.

### Ethanol disrupts convergent extension by disrupting polarized cellular protrusions

The phenotypes observed in ethanol-exposed *vangl2* mutants suggest that ethanol further disrupts convergent extension. Future analyses directly comparing the effects of ethanol and other disruptors of convergent extensions, such as blebbistatin, on filopodial dynamics will aid our understanding of the mechanism of ethanol. Our findings are consistent with previous observations that high doses of ethanol can generate phenotypes consistent with a disruption of convergent extension in zebrafish and Xenopus [16, 17, 51, 52]. More recently, zebrafish microarray analyses demonstrated that disrupted gastrulation movements due to ethanol exposure were caused by a reduction in *pchd18a* expression [53]. The expression of *pchd18a* is not altered in our dataset. However, Sarmah and colleagues used the ethanol-sensitive TL strain and exposed embryos to ethanol from 2 to 8 hpf. This later difference is particularly intriguing given our finding that the time window of greatest sensitivity to ethanol was different for *vangl2* heterozygotes and mutants, with heterozygotes being most sensitive at 3.3 hpf (the earliest time we tested). This raises the interesting possibility that the earlier time window of teratogenesis is due to a different mechanism.

## Conclusions

We propose that ethanol leads to midfacial defects and cyclopia, not by dysregulating Wnt/PCP at the level of transcription, but indirectly through inhibition of convergent extension and mislocalization of important developmental regulators like *shha*. At the start of convergent extension, lateral gastrula cells migrate and converge to the dorsal region of the developing embryo, where they intercalate between neighboring cells to drive extension of the body axis [21, 54, 55]. As these cells begin their dorsal migration, they elongate along their mediolateral axis and polarize their actin-based cytoskeletal processes medially and laterally to drive intercalation [55, 56]. Convergent extension movements during gastrulation also alter the shape of the eye field, whereby diencephalic precursor cells articulate both medially and caudally [57]. We know from previous work that *vangl2* mutant lateral gastrula cells fail to elongate their mediolateral axis and have a slight anterior bias in their dorsal trajectory [40, 58]. In addition to their shape changes, *vangl2* mutant ectodermal cells have more filopodia than wildtype cells and defective polarization (i.e., less filopodia localized on the trailing edge), resulting in a compromised dorsal trajectory [41]. Using live confocal imaging coupled with memGFP expression, we examined the effect of ethanol on filopodia number and polarity in *vangl2* embryos. Interestingly, we did not find more filopodia in *vangl2* lateral mesodermal cells relative to wildtype cells. Despite this, we find that ethanol-treated *vangl2* mutant embryos have more filopodia on their anterior/posterior edge relative to their mediolateral edge. In support of our hypothesis, we demonstrate that ethanol disrupts protrusion polarity, convergent extension, and *shha* localization, with greater disruption observed in *vangl2* mutants.

## Methods

### Zebrafish (*Danio rerio*) care and use

Zebrafish were cared for using standard IACUC-approved protocols at the University of Texas at Austin. The wild-type AB strain was used for RNA-seq analysis because it is commonly used in zebrafish research and has been extensively inbred to minimize genetic heterogeneity. The *vangl2*^*m209*^ allele, originally described as *tri*^*m209*^ (ZIRC Cat# ZL435, RRID:ZIRC_ZL435) [40], was obtained from the Zebrafish International Resource Center (ZIRC) as reported [13]. The gpc4^*fr6*^ line was provided by Dr. Lila Solnica-Krezel. Adult fish were maintained on a 14-h/10-h light-dark cycle at 28.5°C. Embryos were collected and staged according to morphology and somite number [59].

### Chemical treatments

Embryos were treated with 1% ethanol diluted in embryo media, a dose that does not disrupt normal facial development. This dose mimics an acute (binge-like) alcohol exposure roughly equivalent to a blood alcohol concentration of 0.19% in humans. AB embryos were treated with a 50-μM cyclopamine (Toronto Research Chemicals C988400), diluted in embryo media. The concentration of ethanol (vehicle) in the embryo media was controlled for between treatment groups and equals a final concentration of 0.5% in the EM. Blebbistatin (Sigma-Aldrich B0560) was dissolved in DMSO and diluted to a concentration of 37.5 μM in 3 mL of embryo media. Following chemical exposure, embryos were washed 3x in embryo media and then grown to 4 dpf before fixation. Samples sizes for all figures with treatment groups are provided in Additional file 9: Table S2.

### mRNA injection

memGFP cloned in the pCS2 vector was linearized by *NotI* restriction endonuclease. Synthetic mRNA was generated using the mMESSAGE mMACHINE^TM^ SP6 Transcription Kit (Invitrogen, AM1340). Embryos were microinjected at 100pg between the 8 and 16 cell stages [41].

### Sample collection and RNA extraction

Single embryos were manually dechorionated and collected in a 1.75-mL microcentrifuge tube with 500 mL of TRIzol reagent (Life Technologies, 15596-026). Embryos were homogenized with a motorized pestle (VWR, 47747-370) and stored at −80°C until RNA extraction. The total RNA was processed according to the TRIzol RNA isolation protocol. Samples were re-suspended with 50 μL of nuclease-free water and subsequently purified using the RNA Clean & Concentrator kit (Zymo, R1018). The concentration of each sample was determined using a Nanodrop spectrophotometer. The quality of total RNA was analyzed with the Agilent BioAnalyzer to ensure that the RNA Integrity Number (RIN) was ≥ 8. Samples were submitted to the Genomic Sequencing and Analysis Facility (GSAF) at the University of Texas at Austin. The GSAF performed standard RNA-seq library preparations with poly-A mRNA capture.

### Tukey’s honest test

Statistical significance of differences between groups is indicated with compact letter display. Groups with different letters are significantly different from one another (p < 0.05; Tukey’s honest test). Groups that share any letter are not significantly different. For instance, a group labeled “a” is significantly different from one labeled “b.” A group labeled “ab” is not significantly different from one labeled “a” or one labeled “b.” See Additional file 9: Table S2 for all p values.

### RNA-seq data processing

Sequencing on the NextSeq 500 platform produced an average of 40.8 million ± 1.4 million (SE) raw paired-end reads per sample. Adapter trimming was performed using Cutadapt with a minimum length of 25 bp [60]. Following adapter trimming, we retained an average of 40.0 ± 1.3 million (SE) reads per sample. Genome Reference Consortium Zebrafish Build 10 (GRCz10) for *D. rerio* was downloaded from Ensembl [61]. Trimmed reads were mapped to the reference using STAR [62]. The mean mapping efficiency was 78.2% ± 0.8% (SE). Following mapping, PCR duplicates were removed using Picard (https://broadinstitute.github.io/picard/). The duplication rate was estimated at 85% ± 0.8% (SE). Sorting and conversion between SAM and BAM files were performed using samtools [63]. Reads mapping to annotated genes were counted with HTseq version 0.6.1p1 using the intersection nonempty mode [64]. The final number of reads mapped to annotated genes was on average 3.9 ± 0.2 million reads per sample. Detailed instructions and example commands for implementing the data processing steps described above are available on Github (https://github.com/grovesdixon/Drerio_early_ethanol_RNAseq).

### Differential expression analysis

Normalization and statistical analysis of read counts were performed using DESeq2 [65]. Factors included in the differential expression models were *ethanol treatment* (control, treated), *developmental timepoint* (8 hpf, 10 hpf, and 14 hpf), and *sequencing batch* (experiment 1 or experiment 2). Because none of the 6 hpf samples were treated with ethanol, these samples were not included. We tested for differential expression associated with ethanol treatment using likelihood ratio tests—comparing the model including all three factors to a reduced model that did not include ethanol treatment. To further examine stage-specific ethanol effects, we split the samples by developmental timepoint and tested for ethanol effects within each group.

###  Quantitative reverse transcription PCR (RT-qPCR)

To validate our RNA-seq data, we selected two genes to test using RT-qPCR. The total RNA was reverse transcribed using SuperScript™ First-Strand Synthesis System for RT-PCR (Invitrogen) with oligo-d(T) primers. RT-qPCR was performed with Power Sybr Green PCR Master Mix (Thermo Fisher Scientific, 4367659) on the Applied Biosystems ViiA™ 7 Real-Time PCR System. QuantStudio Real-Time PCR Software was used for data analysis using the 2^–∆∆Ct^ method of relative gene expression analysis. The endogenous control *lsm12b* (*like-Sm protein 12 homolog b*) was used based on its stable expression profiles across treatment and stage groups in the RNA-seq dataset.

### KEGG enrichment methods and results

We tested for enrichment of significant upregulated and downregulated genes among KEGG pathways separately using Fisher’s exact tests. For each KEGG pathway, we tested a two-way contingency table with inclusion in the KEGG pathway as columns, and significant (FDR < 0.05) upregulation or downregulation as rows. P values were then adjusted for multiple tests using Benjamini-Hochberg method [66]. In the event that multiple genes from the dataset were annotated with the same pathway member, the log_2_ fold difference for the gene with the greatest absolute value for the difference was used.

Three KEGG pathways were enriched for significant differential expression. Two for upregulated genes and one for downregulated genes. The upregulated KEGG pathways (dre00250 and dre00480) were (1) alanine, aspartate and glutamate metabolism, and (2) glutathione metabolism. The downregulated KEGG pathway (dre03013) was RNA transport. The pathview figures for these are in kegg_pathways/significant_pathway_figures/ in the git repository.

### Cartilage and bone staining and measurements

Embryos were fixed at 4 days post fertilization (dpf) and stained with Alcian blue for cartilage and Alizarin red for mineralized bone [67]. Whole mounts of *vangl2*^*m209*^ embryos were captured using a Zeiss Axio Imager A1 microscope. To assess the degree of cyclopia, the distance between the medial edges of the lenses was measured using the AxiovisionLE software.

### In situ hybridization

Antisense digoxygenin-labeled riboprobes for *shha*, *pax2*, *dlx3*, *six3a*, and *rx3* (together as a gift from Dr. Steve Wilson) were used. Whole-mount in situ hybridization was performed as described [68]. Images were captured using the Zeiss Axio Imager A1, and expression domains were measured using the AxiovisionLE software. An ANOVA and post hoc Tukey’s test were used for statistical analyses.

### Confocal imaging

Control and ethanol-treated (6–10 hpf) embryos were dechorionated and staged at 10 hpf. Homozygous mutants were distinguished between wild-type and heterozygotes by their body axis elongation at the bud stage. Live embryos were mounted in methyl cellulose. Images were acquired using a Zeiss LSM710 confocal microscope using a ×10 and ×60 objective.

### Quantification of filopodia

The number of filopodia was acquired through projections of Z-stacks using the ×60 objective on the Zen software. The angle of projections was calculated using Fiji. Rose diagrams of the mean number of filopodia found were plotted using geom_bar() and the coord_polar() functions from ggplot2. The mean numbers of filopodia for different embryonic regions were compared between groups using ANOVA and Tukey’s “honest significant difference” method. Significantly distinct means were assigned based on Tukey’s honest significant difference results using the R package multcomp [69].

## Supplementary Information

**Additional file 1: Fig. S1.** Age is the major driver of variation in the dataset**.** Heatmap showing overall correlation of gene expression among samples. Samples were hierarchically clustered based on similarity. Treatment [control or ethanol treated (C or E)] and developmental age [hours post fertilization (6, 8, 10, 14, or)] are indicated in the row and column labels.

**Additional file 2: Fig. S2.** There are more upregulated than downregulated genes among ethanol-treated individuals. Volcano plot showing variation in the transcriptional response to ethanol treatment across developmental timepoints. Significant genes (FDR < 0.1) are indicated in red. For each subset, the names of the topmost significantly dysregulated genes are noted near gene’s data point **A** All timepoints combined together **B** 8 hpf only **C** 10 hpf only **D** 14 hpf only.

**Additional file 3: Fig. S3.** WGCNA identifies two modules that are significantly correlated with ethanol exposure. **A** Dendrogram illustrating the hierarchical clustering of the genes and with their corresponding modules colors. The top layer of colors indicates the Dynamic Tree Cutoff, including all assigned modules, before merging by module similarity. The bottom layer indicates the module colors after merging. These merged modules were used for further analysis. **B** Heatmap of module-trait correlations. The eigengene for each module was correlated with ethanol treatment (ethanol), hours post fertilization (age), experimental batch (seqjob), and as a negative control, a randomly shuffled version of the ethanol treatments (rand.eth). Intensity of the color in each cell indicates the strength of correlation between the module (row labels) and the sample trait (column labels). Two modules, (mediumpurple4 and darkolivegreen4) significantly correlated with ethanol treatment (p < 0.05). **C** Scatterplots of correlation with ethanol treatment against module membership. Each datapoint is a gene assigned to the indicated module. Ethanol correlation is the Pearson correlation between the gene’s expression level and ethanol treatment. Module membership is the correlation between the genes expression level and the module eigengene and describes how well the gene matches the overall patterns of the module. **D** Boxplot of log_2_ fold differences due to ethanol for the two significant modules (darkolivegreen4 and mediumpurple4). **E** Changes in gene expression from the RNA-seq were validated using wild-type embryos at 10 hpf. *znf1015* was selected from the darkolivegreen4 module. n=5, for each group (p = 0.0382) and **F** *slc16a9a* was selected from the mediumpurple4 module. n=5, for each group (p < 0.0001). Fold change indicates the degree of change between control and ethanol-treated stage-matched embryos. **G** Gene ontology enrichment tree for Molecular Function for the darkolivegreen4 module. **H** Gene ontology enrichment tree for Molecular Function for the mediumpurple4module.

**Additional file 4: Fig. S4.** Expression of hub genes for development-related modules indicate ethanol did not retard developmental progression. **A-K** Development-related WGCNA modules were identified as those with significant relationship to hours post fertilization (Pearson correlation; p < 0.05). The hub gene for each of these modules was identified as the gene assigned to that module with the highest module membership (defined as the correlation of the gene’s expression level with the module eigengene). Normalized expression levels for the hub genes were plotted against hours post fertilization. The relationship between expression and time is largely consistent between the ethanol treated (E, teal) and control (C, pink) samples. Even for the two modules associated with ethanol treatment (mediumpurple4 and darkolivegreen4), the slopes of the lines are very similar, indicating ethanol exposure did not cause significant developmental delay.

**Additional file 5: Table S1.** Module membership values for all genes in the mediumpurple4 and darkolivegreen4 modules.

**Additional file 6: Fig. S5.** Cell death by TUNEL at 11 hpf in control and ethanol-treated *vangl2* mutants. The number of positive cells in the eye field (indicated by dashed circle) was not higher in ethanol-treated homozygous mutants or their siblings. The genotype *vangl2* ^*+/?*^ denotes *vangl2* heterozygous or wild-type siblings, phenotyped by their elongated body axis relative to the homozygous mutants. ant = anterior; nc = notochord.

**Additional file 7: Fig. S6.** Ethanol alters *rx3* expression in the eye field. Expression pattern of transcription factor *rx3*, stained using whole mount *in situ* hybridization at 12 hpf. The genotype *vangl2*^*+/?*^ denotes *vangl2* heterozygous or wild-type siblings, phenotyped by their elongated body axis relative to homozygous mutants. Dorsal view, anterior to the left.

**Additional file 8: Fig. S7.** Loss of *gpc4* exacerbates cyclopia in a dose-dependent manner. **A** Alcian blue and Alizarin red whole- and flat-mount staining of untreated and 1% ethanol-treated (6 hpf – 4 dpf) *gpc4* homozygous mutants. Embryos fixed at 4 dpf. Dorsal view, anterior to the left. **B** Alcian blue and Alizarin red whole-mount staining of control and 1.6% ethanol-treated (6 hpf – 24 hpf) *gpc4* homozygous mutants. Embryos fixed at 4 dpf. Dorsal view, anterior to the left. Dose-response curve of *gpc4* mutants treated with 1-1.9% ethanol (6-30 hpf). Sample size provided in Additional file 9: Table S2. **C** Percent cyclopia in embryos carrying compound *vangl2;gpc4* mutant alleles. Sample size provided in Additional file 9: Table S2.

**Additional file 9: Table S2.** Sample size and p-values. Sample size for all applicable experiments provided in the first tab of the Excel file. P-value comparisons provided in the second tab of the Excel file.

## Data Availability

All data generated during this study are included in this published article and the supplementary information files. Supplementary sequencing information is available in the github repository under grovesdixon/Drerio_early_ethanol_RNAseq (https://github.com/grovesdixon/Drerio_early_ethanol_RNAseq). The datasets generated and analyzed during the current study are available in the NCBI repository (Accession: PRJNA605033) https://www.ncbi.nlm.nih.gov/bioproject/PRJNA605033 [70].
