# Supplementary figures and images for: Exposure to ethanol leads to midfacial hypoplasia in a zebrafish model of FASD via indirect interactions with the Shh pathway
Source: BMC Biol. 2021 Jul 1;19:134. doi: 10.1186/s12915-021-01062-9 (PMC8247090; doi:10.1186/s12915-021-01062-9)

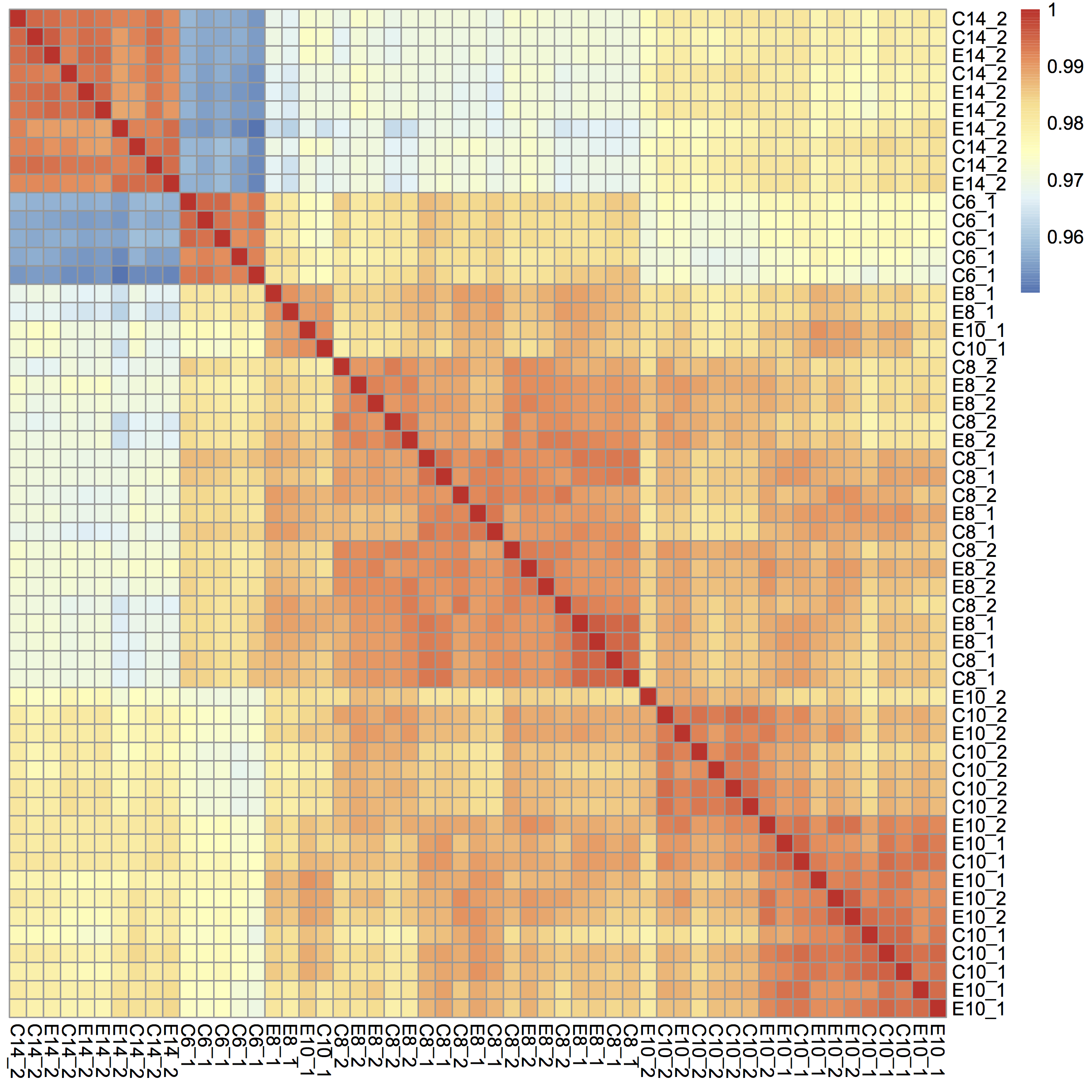

Supplement: Supplementary file 1 — Additional file 1: Fig. S1. Age is the major driver of variation in the dataset. Heatmap showing overall correlation of gene expression among samples. Samples were hierarchically clustered based on similarity. Treatment [control or ethanol treated (C or E)] and developmental age [hours post fertilization (6, 8, 10, 14, or)] are indicated in the row and column labels. [file 12915_2021_1062_MOESM1_ESM.tif]

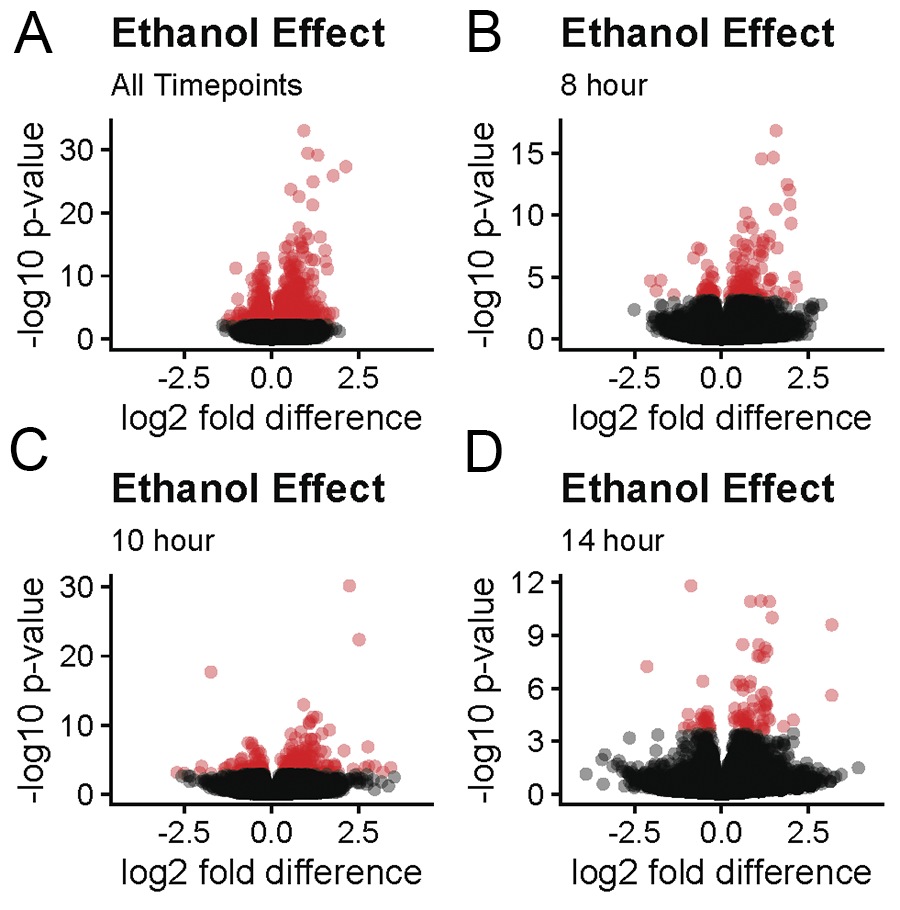

Supplement: Supplementary file 2 — Additional file 2: Fig. S2. There are more upregulated than downregulated genes among ethanol-treated individuals. Volcano plot showing variation in the transcriptional response to ethanol treatment across developmental timepoints. Significant genes (FDR < 0.1) are indicated in red. For each subset, the names of the topmost significantly dysregulated genes are noted near gene’s data point A All timepoints combined together B 8 hpf only C 10 hpf only D 14 hpf only. [file 12915_2021_1062_MOESM2_ESM.tif]

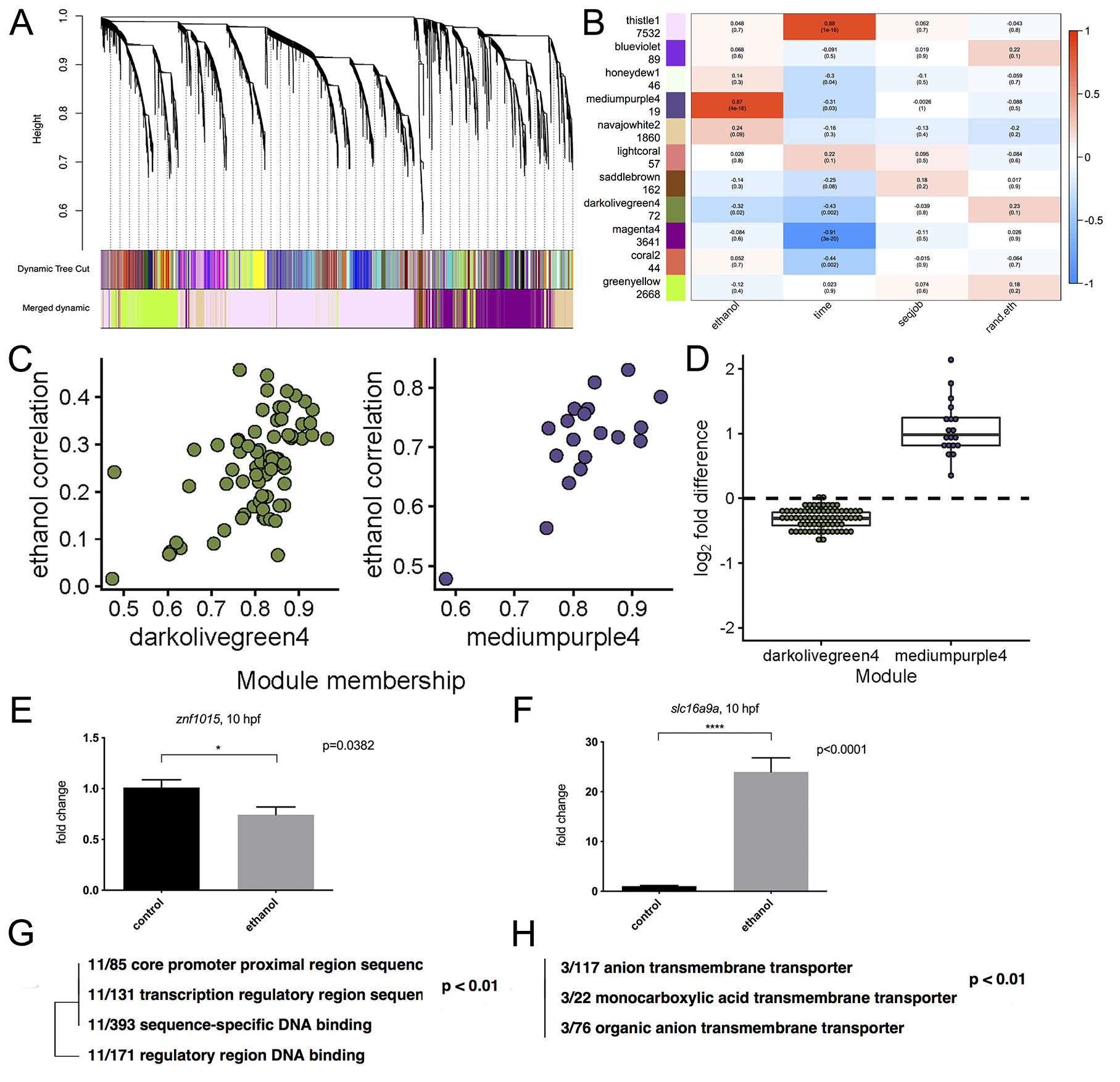

Supplement: Supplementary file 3 — Additional file 3: Fig. S3. WGCNA identifies two modules that are significantly correlated with ethanol exposure. A Dendrogram illustrating the hierarchical clustering of the genes and with their corresponding modules colors. The top layer of colors indicates the Dynamic Tree Cutoff, including all assigned modules, before merging by module similarity. The bottom layer indicates the module colors after merging. These merged modules were used for further analysis. B Heatmap of module-trait correlations. The eigengene for each module was correlated with ethanol treatment (ethanol), hours post fertilization (age), experimental batch (seqjob), and as a negative control, a randomly shuffled version of the ethanol treatments (rand.eth). Intensity of the color in each cell indicates the strength of correlation between the module (row labels) and the sample trait (column labels). Two modules, (mediumpurple4 and darkolivegreen4) significantly correlated with ethanol treatment (p < 0.05). C Scatterplots of correlation with ethanol treatment against module membership. Each datapoint is a gene assigned to the indicated module. Ethanol correlation is the Pearson correlation between the gene’s expression level and ethanol treatment. Module membership is the correlation between the genes expression level and the module eigengene and describes how well the gene matches the overall patterns of the module. D Boxplot of log2 fold differences due to ethanol for the two significant modules (darkolivegreen4 and mediumpurple4). E Changes in gene expression from the RNA-seq were validated using wild-type embryos at 10 hpf. znf1015 was selected from the darkolivegreen4 module. n=5, for each group (p = 0.0382) and F slc16a9a was selected from the mediumpurple4 module. n=5, for each group (p < 0.0001). Fold change indicates the degree of change between control and ethanol-treated stage-matched embryos. G Gene ontology enrichment tree for Molecular Function for the darkolivegreen4 module. H Gen [file 12915_2021_1062_MOESM3_ESM.tif]

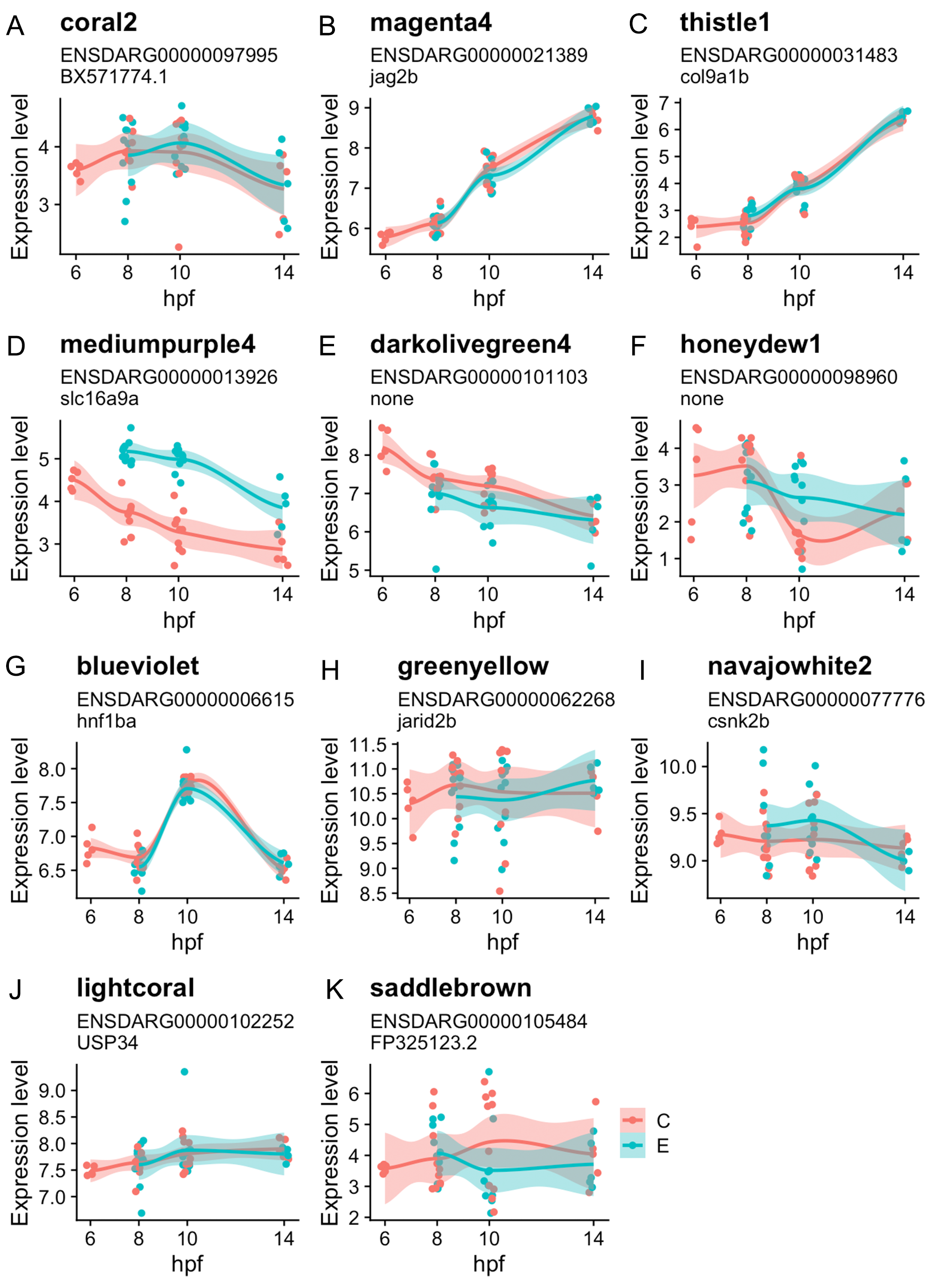

Supplement: Supplementary file 4 — Additional file 4: Fig. S4. Expression of hub genes for development-related modules indicate ethanol did not retard developmental progression. A-K Development-related WGCNA modules were identified as those with significant relationship to hours post fertilization (Pearson correlation; p < 0.05). The hub gene for each of these modules was identified as the gene assigned to that module with the highest module membership (defined as the correlation of the gene’s expression level with the module eigengene). Normalized expression levels for the hub genes were plotted against hours post fertilization. The relationship between expression and time is largely consistent between the ethanol treated (E, teal) and control (C, pink) samples. Even for the two modules associated with ethanol treatment (mediumpurple4 and darkolivegreen4), the slopes of the lines are very similar, indicating ethanol exposure did not cause significant developmental delay. [file 12915_2021_1062_MOESM4_ESM.tif]

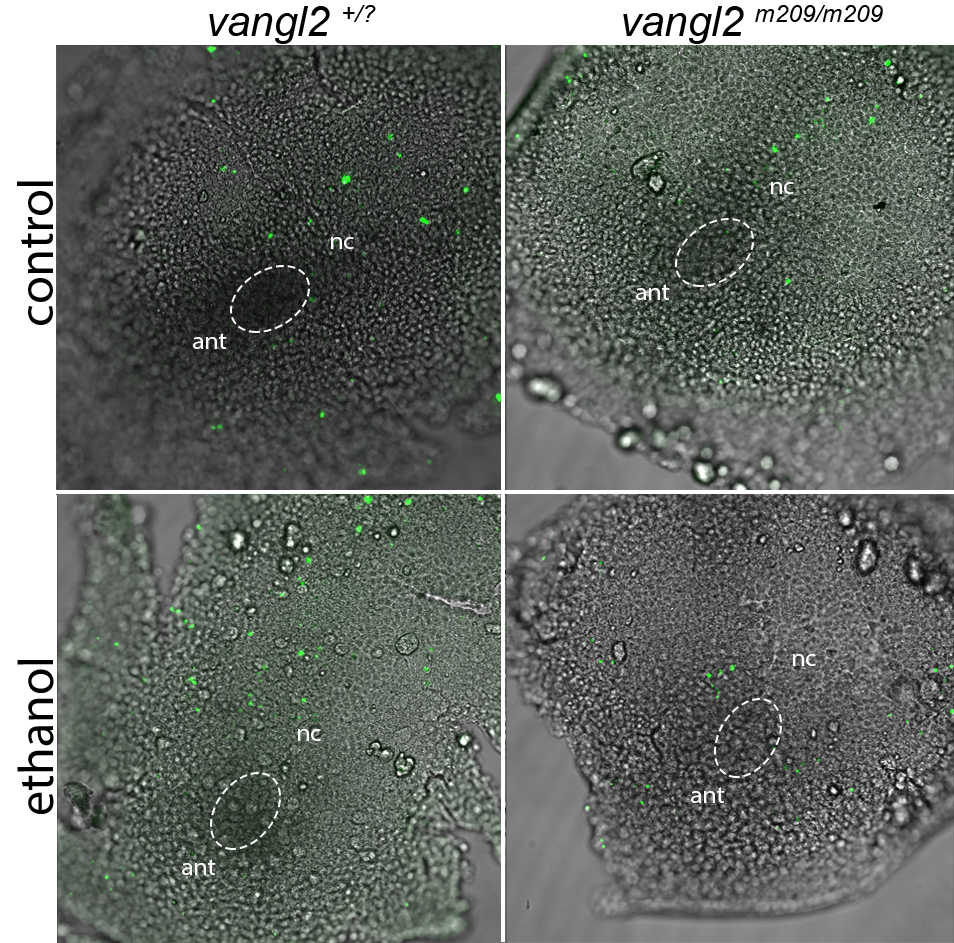

Supplement: Supplementary file 6 — Additional file 6: Fig. S5. Cell death by TUNEL at 11 hpf in control and ethanol-treated vangl2 mutants. The number of positive cells in the eye field (indicated by dashed circle) was not higher in ethanol-treated homozygous mutants or their siblings. The genotype vangl2 +/? denotes vangl2 heterozygous or wild-type siblings, phenotyped by their elongated body axis relative to the homozygous mutants. ant = anterior; nc = notochord. [file 12915_2021_1062_MOESM6_ESM.tif]

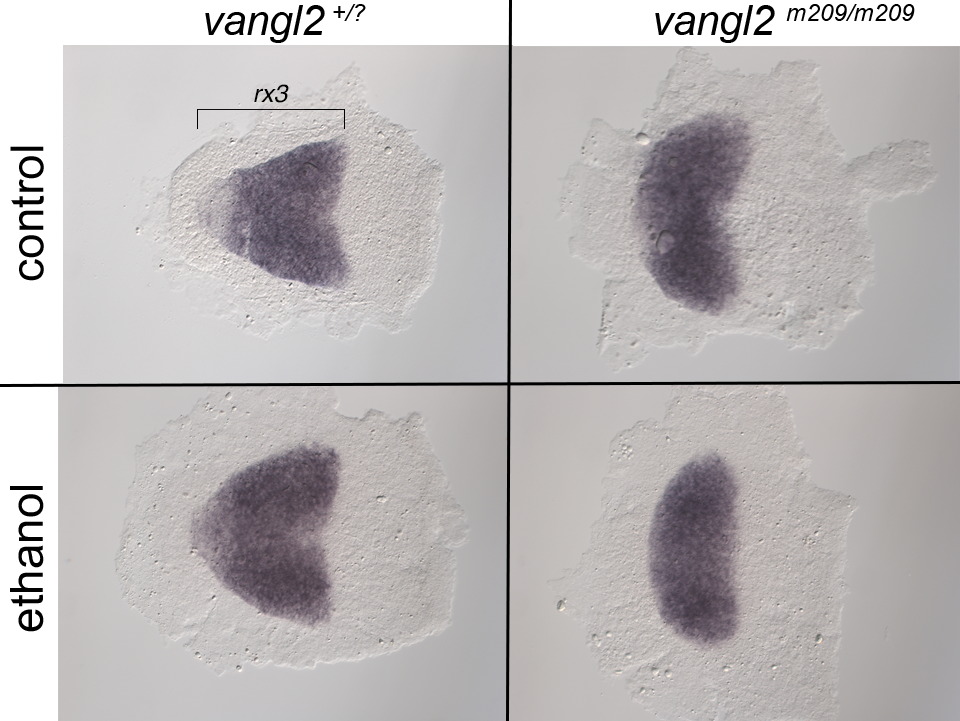

Supplement: Supplementary file 7 — Additional file 7: Fig. S6. Ethanol alters rx3 expression in the eye field. Expression pattern of transcription factor rx3, stained using whole mount in situ hybridization at 12 hpf. The genotype vangl2+/? denotes vangl2 heterozygous or wild-type siblings, phenotyped by their elongated body axis relative to homozygous mutants. Dorsal view, anterior to the left. [file 12915_2021_1062_MOESM7_ESM.tif]

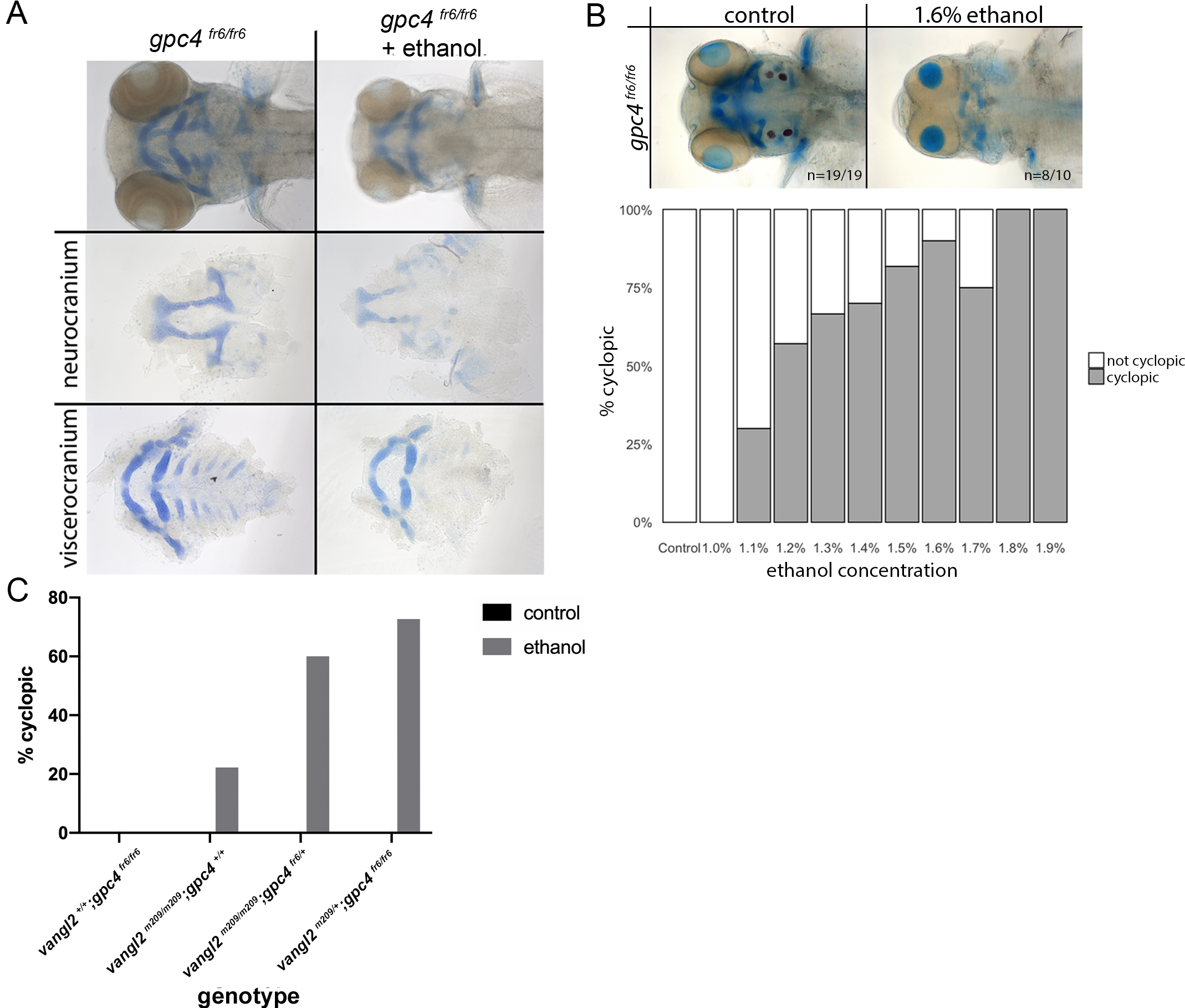

Supplement: Supplementary file 8 — Additional file 8: Fig. S7. Loss of gpc4 exacerbates cyclopia in a dose-dependent manner. A Alcian blue and Alizarin red whole- and flat-mount staining of untreated and 1% ethanol-treated (6 hpf – 4 dpf) gpc4 homozygous mutants. Embryos fixed at 4 dpf. Dorsal view, anterior to the left. B Alcian blue and Alizarin red whole-mount staining of control and 1.6% ethanol-treated (6 hpf – 24 hpf) gpc4 homozygous mutants. Embryos fixed at 4 dpf. Dorsal view, anterior to the left. Dose-response curve of gpc4 mutants treated with 1-1.9% ethanol (6-30 hpf). Sample size provided in Additional file 9: Table S2. C Percent cyclopia in embryos carrying compound vangl2;gpc4 mutant alleles. Sample size provided in Additional file 9: Table S2. [file 12915_2021_1062_MOESM8_ESM.tif]
